# New global analysis of the microRNA transcriptome of primary tumors and lymph node metastases of papillary thyroid cancer

**DOI:** 10.1186/s12864-015-2082-3

**Published:** 2015-10-21

**Authors:** Manuel Saiselet, David Gacquer, Alex Spinette, Ligia Craciun, Myriam Decaussin-Petrucci, Guy Andry, Vincent Detours, Carine Maenhaut

**Affiliations:** IRIBHM, Université libre de Bruxelles, 808 route de Lennik, B-1070 Brussels, Belgium; Tumor Bank of the J. Bordet Cancer Institute, 1000 Brussels, Belgium; Service d’anatomie et cytologie pathologiques, Centre Hospitalier Lyon Sud, 69495 Pierre Benite Cedex, France; J. Bordet Cancer Institute, Surgery Department, 1000 Brussels, Belgium; Welbio, Université libre de Bruxelles, Brussels, Belgium

**Keywords:** microRNA, miRNome, Papillary, Thyroid, Cancer, Metastasis, isomiR

## Abstract

**Background:**

Papillary Thyroid Cancer (PTC) is the most prevalent type of endocrine cancer. Its incidence has rapidly increased in recent decades but little is known regarding its complete microRNA transcriptome (miRNome). In addition, there is a need for molecular biomarkers allowing improved PTC diagnosis.

**Methods:**

We performed small RNA deep-sequencing of 3 PTC, their matching normal tissues and lymph node metastases (LNM). We designed a new bioinformatics framework to handle each aspect of the miRNome: whole expression profiles, isomiRs distribution, non-templated additions distributions, RNA-editing or mutation. Results were validated experimentally by qRT-PCR on normal samples, tumors and LNM from 14 independent patients and in silico using the dataset from The Cancer Genome Atlas (small RNA deepsequencing of 59 normal samples, 495 PTC, and 8 LNM).

**Results:**

We performed small RNA deep-sequencing of 3 PTC, their matching normal tissues and lymph node metastases (LNM). We designed a new bioinformatics framework to handle each aspect of the miRNome: whole expression profiles, isomiRs distribution, non-templated additions distributions, RNA-editing or mutation. Results were validated experimentally by qRT-PCR on normal samples, tumors and LNM from 14 independent patients and *in silico* using the dataset from The Cancer Genome Atlas (small RNA deep-sequencing of 59 normal samples, 495 PTC, and 8 LNM). We confirmed already described up-regulations of microRNAs in PTC, such as miR-146b-5p or miR-222-3p, but we also identified down-regulated microRNAs, such as miR-7-5p or miR-30c-2-3p. We showed that these down-regulations are linked to the tumorigenesis process of thyrocytes. We selected the 14 most down-regulated microRNAs in PTC and we showed that they are potential biomarkers of PTC samples. Nevertheless, they can distinguish histological classical variants and follicular variants of PTC in the TCGA dataset. In addition, 12 of the 14 down-regulated microRNAs are significantly less expressed in aggressive PTC compared to non-aggressive PTC. We showed that the associated aggressive expression profile is mainly due to the presence of the BRAF V600E mutation. In general, primary tumors and LNM presented similar microRNA expression profiles but specific variations like the down-regulation of miR-7-2-3p and miR-30c-2-3p in LNM were observed. Investigations of the 5p-to-3p arm expression ratios, non-templated additions or isomiRs distributions revealed no major implication in PTC tumorigenesis process or LNM appearance.

**Conclusions:**

Our results showed that down-regulated microRNAs can be used as new potential common biomarkers of PTC and to distinguish main subtypes of PTC. MicroRNA expressions can be linked to the development of LNM of PTC. The bioinformatics framework that we have developed can be used as a starting point for the global analysis of any microRNA deep-sequencing data in an unbiased way.

**Electronic supplementary material:**

The online version of this article (doi:10.1186/s12864-015-2082-3) contains supplementary material, which is available to authorized users.

## Background

Thyroid cancer is the most prevalent type of endocrine cancer and papillary thyroid cancer (PTC) is, by far, the most frequent form 40 to 60 % patients present lymph node metastases (LNM). These metastases increase the risk of recurrence. The rate of mortality is low but the presence of LNM decreases long term survival, mainly for older patients (>45 years) [1, 2]. The diagnostic process is mainly based on the cytological profile of the tissue performed on a biopsy of the thyroid nodule. However, up to 30 % of the preoperative biopsies are inconclusive [3, 4]. The lobectomy or total thyroidectomy is then frequently proposed even if the probability of a malignant nodule is low and the surgery risky [5, 6]. There is thus a need for molecular biomarkers to avoid unnecessary surgery and to personalize treatment. microRNAs are among the most studied potential biomarkers of thyroid cancers [7, 8]. In addition, the implication of microRNAs in the aggressiveness of PTC and LNM formation is poorly known.

MicroRNAs are a class of small non-coding RNAs, 19 to 25 nucleotides long, which act on the stability and the translation efficiency of their target mRNAs [9]. It has been described that each microRNA may control the expression of up to several hundreds of genes, involved in almost all programs of cell biology [10]. The intermediate precursor (pre-miR) form is composed by a double-stranded duplex consisting of two arms (formerly known as miR/miR* but now referred as 5p-arm/3p-arm) and a terminal stem loop. In some cases, it has been shown that both arms can produce a functional mature microRNA [11]. An expression ratio of the amount of the 5p-arm to the 3p-arm can then be calculated.

MicroRNAs may exist under different isoforms in the cells. These forms may result from alternative cleavages of the precursor during the maturation process. This leads to the coexistence, in individual cell types, of a diversity of sequence lengths, called isomiRs, with a variation of one or several bases at the 5′ end or the 3′ end of the microRNA [12]. Furthermore, some sequence variations may also result from one or several nucleotide additions occurring after cleavage of the precursor. These supplemental nucleotides (mainly A or U bases) are added by nucleotidyl transferases and are generally found at the 3′ end of the mature microRNA. These additions create very particular isomiRs which are called non-templated additions (NTA). NTA generally induce a divergence between the microRNA sequence and its corresponding genomic sequence. SNPs also produce variations in microRNA sequences but their frequency in microRNAs is very low compared to the frequency of SNPs observed across the whole human genome [12]. A-to-I RNA-editing involves the hydrolytic deamination of adenosine to inosine in double-stranded RNA. Since pre-miR form double-stranded duplexes, the editing machinery can modify microRNA sequences as well [13].

These different mechanisms of isoform diversification may co-exist for the same microRNA and the isoform profile of each microRNA may change between cell types. Changes in the 3′ part of the microRNAs are widely the most found, most of the time they do not lead to a modification of the function of the microRNAs [12, 14]. However, changes in the 5′ may drastically modify the selection of the mRNA targets [15].

It has been shown that microRNA expressions can discriminate human tumor samples from normal samples in different types of cancers [16]. Many studies have shown that microRNAs are involved in the tumorigenesis and the metastatic process of many types of human cancers [17–19]. In particular, for thyroid cancers, numerous modulated microRNAs between normal and tumor samples have been identified [20]. Many of these studies were performed with quantitative RT-PCR or microarrays. These methodologies are broadly used but are based on primer or probe specificity and can miss some expressions due to a lowest affinity for isoforms of the same mature microRNA. Small RNA deep-sequencing offers a more efficient way to study every aspect of the microRNA biogenesis [21, 22]. Some deep-sequencing studies suggest that NTA, isomiRs, 5p-to-3p arm expression ratio or A-to-I editing could be involved in the tumorigenesis of different cancer types [23–28] but only one study analyzed the isomiR distribution in PTC [27]. Small RNA deep-sequencing analysis is often restricted by the necessity of bioinformatics analyses flexible enough to explore every aspect of the miRNome and to decrease technical biases [22, 29]. So far, most of the small RNA deep-sequencing studies used a “homemade” analysis method and there is no “gold standard” procedure.

In our study, we searched for modulated microRNAs in PTC and in LNM samples. We performed small RNA deep-sequencing of a first set of samples composed by 3 PTC, their matching normal tissues and LNM. The samples presented a weak (or lack of) contamination by normal cells, lymphocytes and fibroblasts. We collected and applied all the specific guidelines to design a dedicated bioinformatics framework for the analysis of each aspect of the miRNome from small RNA deep-sequencing data. The results were validated on about 600 independent samples: experimentally by qRT-PCR on normal samples, tumors and LNM from 14 independent patients and *in silico* using the data from The Cancer Genome Atlas (small RNA deep-sequencing data already processed of 495 PTC, 59 normal samples and 8 LNM) [30]. We analyzed all miRNome variations of the samples in order to identify new strong and common biomarkers that might be related to tumorigenesis of the thyrocytes and LNM formation.

## Methods

### Tissue collection

Normal tissues, adjacent tumors and LNM were collected from 17 patients with a PTC diagnosis, confirmed by anapathological analyses. Samples were obtained from: « Service d’anatomopathologie, Institut J Bordet » (Brussels, Belgium) and « Service d’anatomie et de cytologie pathologiques, Centre de Biologie Sud, Centre Hospitalier Lyon Sud » (Lyon, France). This study was approved by the ethics committees of Institut J Bordet and Centre Hospitalier Lyon Sud. Written informed consent was obtained from all participants involved in the study. Tissues were immediately dissected, placed on ice, snap-frozen in liquid nitrogen and stored at −80 °C until RNA processing. All samples were stained by hematoxylin and eosin and their cellular composition was established by a pathologist. The presence of TAM (Tumor Associated Macrophages) was established using a CD163 staining [31]. Cellular composition was estimated in terms of percentage of tumor cells, percentage of adjacent non-tumor cells, lymphocytes and fibrosis infiltrations. Both tumor and metastatic samples were included in the study only if they contained at least 70 % of cancer cells. 3 PTC (classical form) and matched samples were selected for human small RNA deep-sequencing, microdissection, quantitative RT-PCR confirmation and Sanger sequencing (Table 1). 14 PTC (10 classical forms, 3 follicular forms and 1 diffuse sclerosis variant) and matched samples were used for quantitative RT-PCR validation. Main clinical and pathological information are described in Additional file 1: Table S1.

**Table 1:** Cellular composition of the deep-sequenced samples

Patient ID	Sample type	% of tumor cells	% of fibrosis	% of lymphatic cells
1	Normal	0	0	0
1	Tumor	75	0	0
1	Metastasis	80	0	10
2	Normal	0	0	0
2	Tumor	70	30	0
2	Metastasis	90	0	5
3	Normal	0	10	0
3	Tumor	70	0	0
3	Metastasis	80	10	10

All the samples showed a very low (<5 %) CD163 staining, reflecting a very low macrophage infiltration.

### RNA and DNA extraction

Total RNA was extracted from tissues using Qiazol, followed by purification on miRNeasy columns (Qiagen) according to the manufacturer’s recommendations. Genomic DNA was extracted from the remaining mix of phenol-chloroform using homemade protocol described in Additional file 2.

Laser capture microdissection, qRT-PCR and other experimental procedures are described in Additional file 2.

### Human small RNA deep-sequencing: mapping strategy and downstream analyses

Small RNA deep-sequencing of the 3 PTC and their matched samples were performed by the Beijing Genomics Institute (BGI) using the TruSeq Small RNA Sample Prep Kit (Illumina). 24 to 31 million of clean reads per sample were obtained. Illumina reads collapsed by sequence in fasta format are available from NCBI’s Gene Expression Omnibus [32] and are accessible through GEO series accession number GSE57780. In the following sections and in Additional file 2 we describe the bioinformatics pipeline that we used to analyze our small RNA deep-sequencing data and the already processed validation data from the TCGA.

**microRNA reads mapping:** from now on, we will refer to a microRNA tag as a subset of individual Illumina reads having identical sequences and that were collapsed together during data preprocessing. A tag is then defined by its DNA sequence and a count value corresponding to the number of Illumina reads sharing this sequence. To lower computational efforts, tags instead of individual reads were mapped to the specified reference. This increased alignment speed by several orders of magnitude. For the rest of this section, read mapping and tag mapping are equivalent expressions.

There is so far no gold standard method for microRNA reads mapping. Whether reads are mapped on the whole reference genome, known microRNA precursors or even mature microRNAs varies from one study to another, as for the number of allowed mismatches. Nonetheless, specific issues relative to the nature of small RNA deep-sequencing are frequently pointed out and should be considered:

- additions of non-templated nucleotides (NTA) will naturally introduce bases that differ from the reference mainly at the 3′ end of mature microRNA. Short read mappers do not distinguish NTA from “inner” mismatches that are due to SNPs, sequence variation or sequencing errors and this further leads to ambiguous alignments. This problem, referred as cross-mapping [29], occurs mainly for microRNAs appearing in families that differ only by one or two nucleotides;
- the presence of multi-loci microRNAs (such as let-7a-1, let-7a-2, let-7a-3) complicates the definition of unique match. Such tags cannot be excluded on the simple basis that they map equally to multiple places, since the mature microRNA itself can be transcribed from multiple genomic loci;
- cross-mapping issues may also appear when tags are aligned directly to microRNA precursors instead of the whole reference genome [22]. Because small RNA libraries also contain other short RNAs (snRNAs, tRNAs…), these could be mistaken with mature microRNAs having a similar sequence.

**Fig. 1:**
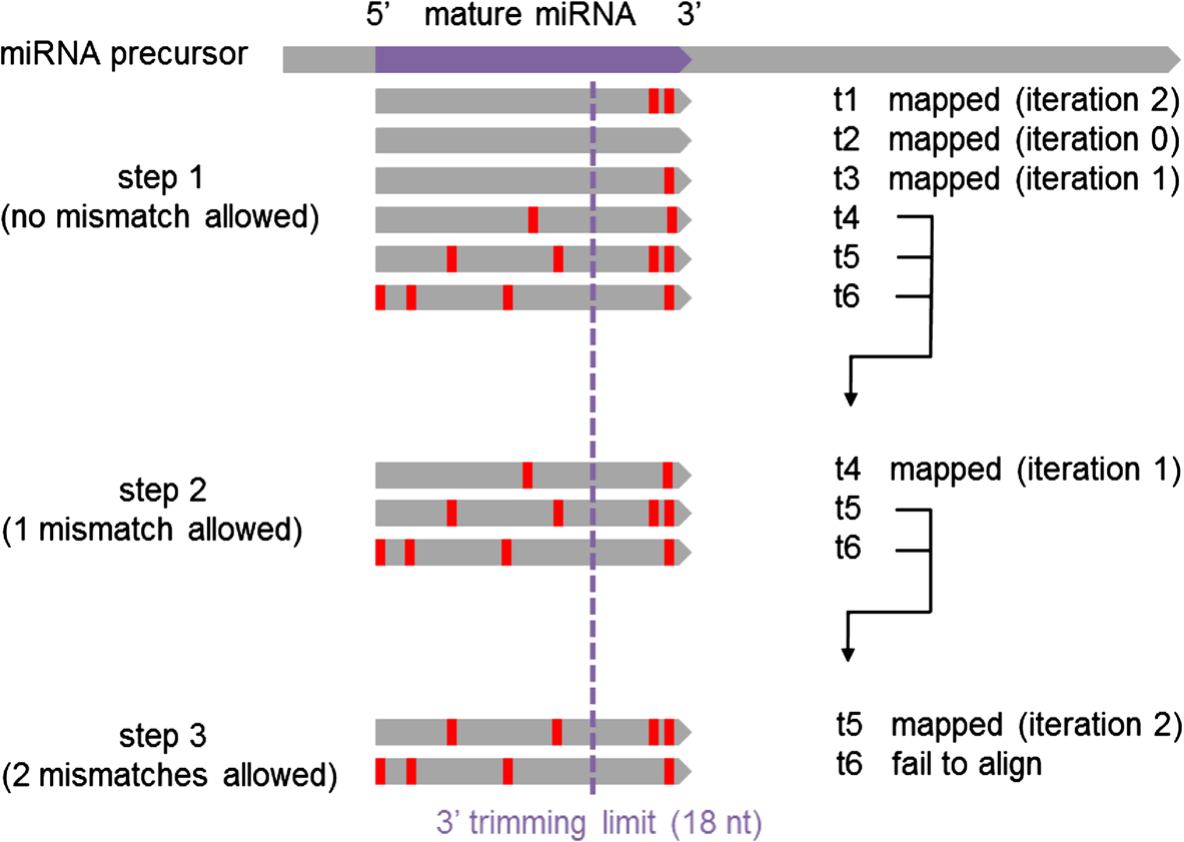
Overview of the stratified alignment strategy used to map short Illumina tags to human pre-microRNAs or whole genome. Three steps allowing respectively 0, 1 and 2 mismatches were used. In each step, tags were iteratively trimmed at the 3′ end until hits could be found with respect to the number of allowed mismatches. A tag was mapped at iteration *i* if *i* nucleotides were removed at its 3′ end before hits could be found. Trimmed tags that could not be mapped before they reached the minimum length of 18 bp were forwarded to the next step where the number of allowed mismatches was gradually increased. This ensured that tags with mixed 3′ addition and genomic variation, such as t4, will show only the real genomic variation when aligned on human precursors or whole genome

We designed a multi-step reads mapping procedure illustrated in Fig. 1 to address these issues. It aims at distinguishing variations due to NTA from “inner” mismatches by trimming tags iteratively until a match can be found. The number of allowed mismatches is gradually increased at each alignment step so that perfect matches are always preferred. Alignment was performed with the Bowtie v0.12.7 aligner [33] setting the –v argument to the desired maximum number of allowed mismatches. During the first step, no mismatches are allowed and reads that fail to align to the specified reference are iteratively trimmed at their 3′ end until a perfect match can be found, or a specified limit is reached (either in terms of minimum length or maximum number of trimmed bases). Then, all tags that could not be perfectly mapped are used as input for the second step, in which one mismatch is allowed this time. When passed to the next step, reads are reverted to their original length, prior any trimming at their 3′ end. The same iterative alignment is applied, so that if a read has one inner mismatch and one mismatch due to 3′ addition, it will be mapped with one mismatch only, after trimming of the latter 3′ addition. Similarly, additional steps can be added with increased number of mismatches. For each mapped tag, we required both its 5-prime and 3-prime coordinate to be located within a maximum of 5 base pairs of its corresponding canonical microRNA coordinates. The size of these windows was chosen so that most isomiR variation could be captured while excluding potential artifacts.

We first applied this strategy using known microRNAs precursors (miRBase v19) as reference. Repeats were kept but tags that were mapped on the opposite strand of known pre-microRNA were considered incorrect and removed. We then used the full reference genome (hg19) as reference with three additional restrictions: (1) only a maximum of one mismatch was allowed; (2) a maximum of 2 bases were trimmed for each tag to avoid excessive length reduction; and (3) repeats were kept only if all matches corresponded to multiple loci of the same microRNA (e.g. multiple matches on let-7a-1, let-7a-2, let-7a-3 were all kept, matches on miR-548a, miR-548b, miR-548e were all discarded). With this strategy, up to 93 % of individual reads could be mapped during the first step when using the human genome hg19 as reference.

**microRNA differential expression profiles:** after aligning reads to the reference genome, we counted the number of mapped reads for both annotated and novel mature microRNAs. Expression analysis was performed using edgeR [34] distributed as an R [35] package available at Bioconductor [36]. As advised in the documentation, we first extracted all microRNAs with a normalized expression (in CPM: Count Per Million mapped reads) of 1 CPM in at least 3 samples, leading to a total of 398 microRNAs that were used as input for differential expression analysis. We used a generalized linear model and designed our analysis so that 3 replicates were available per condition while retaining the paired information for tissue types collected from the same patient. This was done to remove baseline differences between patients when comparing microRNA expressions between conditions (e.g tumor versus normal samples, LNM versus normal samples and LNM versus tumor). By default, edgeR estimates the original size of each library by summing read counts across all genes for a given sample. Because let-7 microRNAs are highly expressed and appear as multi-loci microRNAs (e.g. hsa-let-7a-1, hsa-let-7a-2 and hsa-let-7a-3), several millions of reads were counted multiple times and library sizes were highly over-estimated. Therefore, we set library sizes to the total number of individual sequences present in fasta files for each sample prior computing normalization factors using edgeR calcNormFactors function. Multidimensional scaling analysis and hierarchical clustering were performed in R, Spearman correlations were performed in GaphPad Prism.

## Results

### Down-regulated microRNAs are potential biomarkers of main subtypes of papillary thyroid cancers and associated lymph node metastases

- ***▪*microRNA deep-sequencing data**

**Fig. 2:**
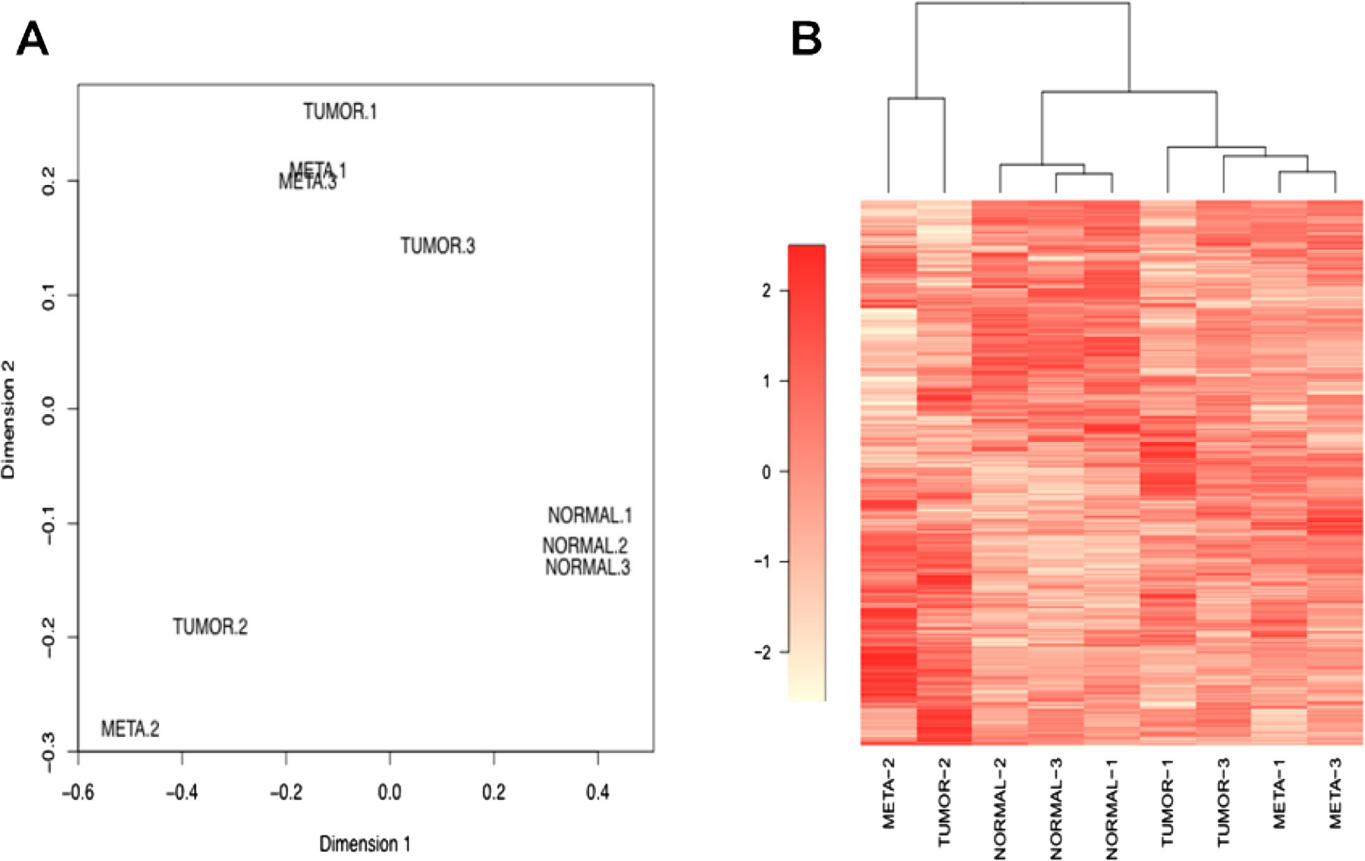
Multi-Dimensional Scaling (**a**) and hierarchical clustering (**b**) of microRNA expression profiles for the deep-sequenced samples. Normal samples, primary tumors and LNM from 3 patients were analyzed by small RNA deep-sequencing. Analyses were performed using 398 microRNAs having a CPM ≥ 1 in at least 2 samples

We analyzed small RNA deep-sequencing expression profiles of the first sample set (three PTC primary tumors (classical variants) and their matched normal adjacent tissues and LNM). These samples were carefully selected on the basis of their normal or tumor cellular composition (Table 1). Multidimensional scaling analysis showed that global microRNA expression patterns of normal samples were close to each other while tumor samples were similar to their related lymph node metastases (Fig. 2a). Tumor and metastasis samples of patient 2 were distant from tumor and metastasis samples of patients 1 and 3. This can be explained by the fact that patients 1 and 3 are both young women, whereas patient 3 is an old man presenting a larger tumor at the time of surgery (Additional file 1: Table S1). These results were confirmed by a hierarchical clustering (Fig. 2b).

**Fig. 3:**
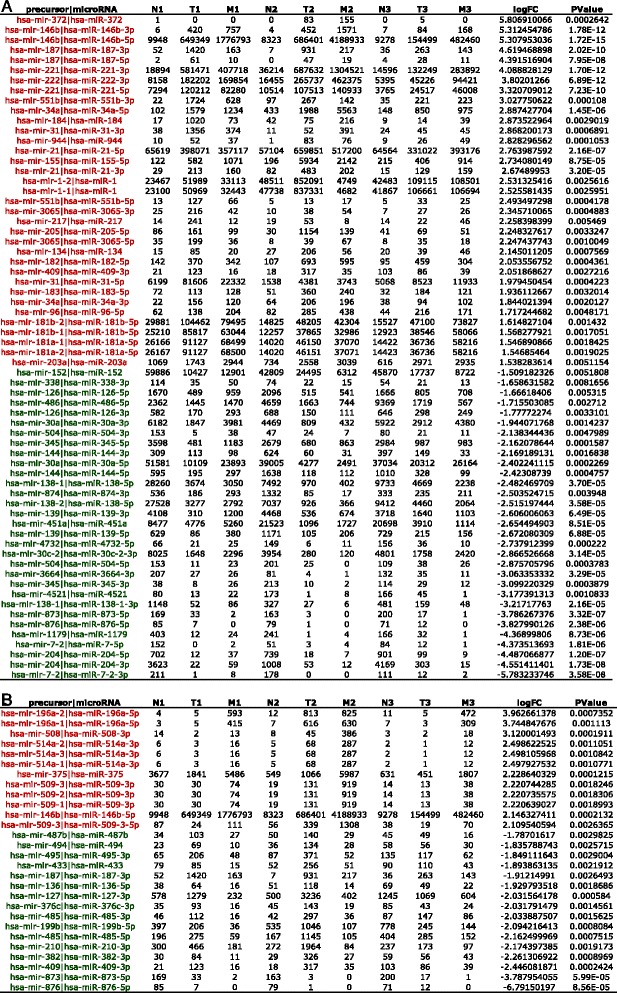
Significant modulated microRNA expressions between samples types in the small RNA deep-sequencing data. Normal samples (N), primary tumors (T) and LNM (M) from 3 patients (M) were analyzed. The read counts are described for each sample. The fold change (FC) is the log 2 expression ratio in CPM. We used a generalized linear model and designed our analysis so that 3 replicates were available per condition while retaining the paired information for tissue types collected from the same patient (See Methods). **a**: comparison between tumors and normal samples. Up-regulated microRNA in tumors are in red and down-regulated microRNAs in tumors are in green. **b**: comparison between LNM and tumor samples. Up-regulated microRNAs in metastases are in red and down-regulated microRNAs in metastases samples are in green

We identified microRNAs differentially expressed between tissue types. We found 32 up-regulated and 30 down-regulated microRNAs in PTC compared to normal samples. As expected, we found well characterized up-regulated microRNAs in PTC (miR-146b-5p, miR-221… [8, 20]), but also significantly down-regulated microRNAs in PTC compared to normal samples (Fig. 3a). Furthermore, we found significant modulations between primary tumors and LNM (Fig. 3b). Small RNA deep-sequencing analysis also identified novel mature microRNAs on known precursors, but none of them appeared to be differentially expressed between tissue types (Additional file 1: Table S2).

- ***▪Experimental validation***

We decided to focus our validations on the newly discovered down-regulated microRNAs in PTC and on microRNAs modulated between tumors and LNM. We used quantitative RT-PCR assays for 20 microRNAs selected from the most down-regulated microRNAs in tumors compared to normal samples (14 assays): miR-1179, miR-7-2-3p, miR-7-5p, miR-876-5p, miR-204-5p, miR-138-3p, miR-138-5p, miR-30c-2-3p, miR-139-3p, miR-139-5p, miR-451a, miR-504, miR-152, miR-873-5p (Fig. 3a) and from the most up- and down-regulated microRNAs in LNM compared to tumors (7 assays): the down-regulated miR-873-5p, miR-876-5p, miR-199b-5p and the up-regulated miR-375, miR-196a-5p, miR-509-3p, miR-509-5p (Fig. 3b). miR-873-5p and miR-876-5p were selected by both criteria. The well-known up-regulated microRNA in PTC miR-146b-5p was used as a control.

**Fig. 4:**
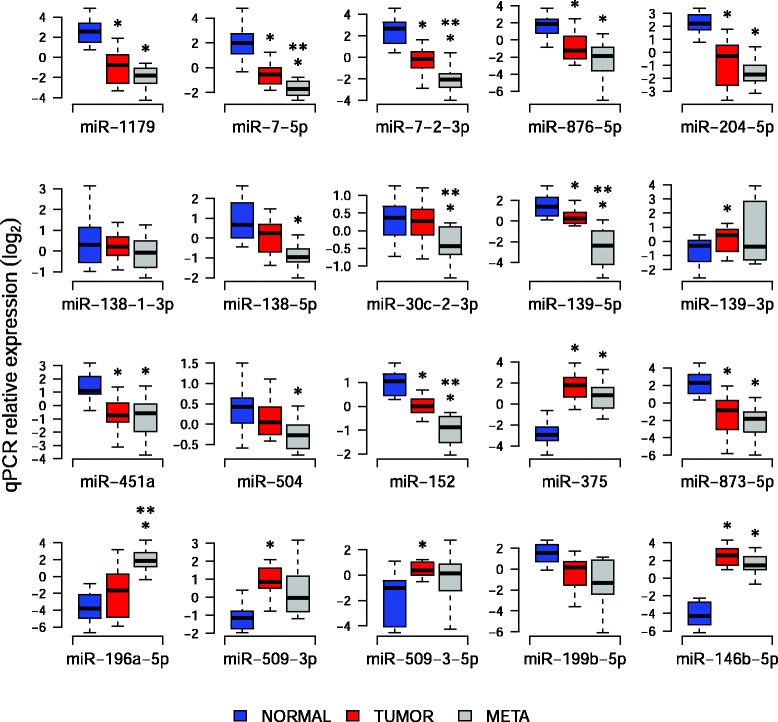
qRT-PCR validation of a selection of differentially expressed microRNAs. Validation was performed on independent samples from 14 patients. Significance of differential expression between tissue types was assessed using a paired *t*-test. *: significant modulation (*p* < 0.05) of expression between normal samples and tumors or LNM. **: significant modulation of expression between tumor samples and LNM. Relative quantification was calculated with the Pfaffl method and normalized between samples based on U6 SnRNA expression

We analyzed the expression profiles of the 20 microRNAs in each deep-sequenced sample (Additional file 1: Figure S1). We found a significant correlation between fold changes (tumor/normal or metastasis/tumor) obtained by deep-sequencing and by qRT-PCR experiments (Spearman r = 0.64, *P* = 0.0023). This result confirmed the utility of qRT-PCR as validation tool. To further validate our deep-sequencing results, we performed qRT-PCR experiments on matched normal, primary tumor and LNM samples from an independent set of 14 patients (Additional file 1: Table S1). Like for deep-sequenced samples, only normal samples with no detectable tumor cells and tumors or metastases with at least 70 % of tumor cells were considered (see Methods). Among the 14 microRNAs downregulated in tumors, we confirmed 9 significant decreases: miR-1179, miR-7-5p, miR-7-2-3p, miR-876-5p, miR-204-5p, miR-139-5p, miR-451a, miR-152 and miR-873-5p (Fig. 4). Although not significantly down-regulated in tumors compared to normal samples, miR-138-5p, miR-30c-2-3p and miR-504 showed nevertheless a decreased expression. Interestingly, some down-regulations were even more pronounced in the LNM and showed significant differences between tumors and LNM: miR-7-5p, miR-7-2-3p, miR-30c-2-3p, miR-139-5p and miR-152. Among the 7 microRNAs differentially expressed between LNM compared to primary tumors, only miR-196a-5p showed a significant up-regulation in the LNM samples, and no modulation between tumors and normal tissues. Although not significantly regulated in the LNM compared to the tumors, miR-375, miR-509-3p and miR-509-3-5p were nevertheless up-regulated and miR-873-5p and miR-876-5p down-regulated in the tumors compared to normal samples. The strong up-regulation of miR-146b-5p in both tumors and LNM is in accordance with previous data (Fig. 4).

To confirm the specific thyroid cell expression of the modulated microRNAs, we performed Laser Captured Microdissection (LCM) on frozen sections from the deep-sequenced samples, and we analyzed the expression profiles of the 20 microRNAs by qRT-PCR in the normal and tumor thyroid cells. Expression variations identified by deep-sequencing, especially for the down-regulated microRNAs in tumors (miR-1179, miR-7-5p, miR-7-2-3p, miR-204-5p, miR-873-5p and miR-876-5p) and the up-regulation of miR-196a-5p in LNM, were detected in the microdissected samples as well (Additional file 1: Figure S1). In addition, we found a significant correlation between fold changes (tumor/normal or metastasis/tumor) obtained by qRT-PCR on deep-sequenced samples and on the microdissections of these samples (Spearman r = 0.63, *P* = 0.0045).

These results on a small set of samples suggest that some of the down-regulated microRNAs are potential biomarkers of PTC tumorigenesis and lymph node metastasis formation and that miR-196a-5p could be related to lymph node metastasis formation.

- ***▪ In silico*validation on the TCGA small RNA deep-sequencing dataset**

In order to further validate our results on the largest set of independent PTC samples, we collected the TCGA public small RNA deep-sequencing data processed by the Broad Institute with an independent bioinformatics approach (see Additional file 2 for a complete description of collected datasets). The microRNA expression profiles of 59 normal samples, 495 primary tumors of all histological subtypes and 8 LNM were available. We used Table S2 from the recent TCGA publication on PTC [30] to obtain the clinical and pathological characteristics of samples. We analyzed the differential expression of the 20 microRNAs previously investigated by qRT-PCR.

#### Down-regulated microRNAs are biomarkers of PTC tumorigenesis and LNM formation

**Fig. 5:**
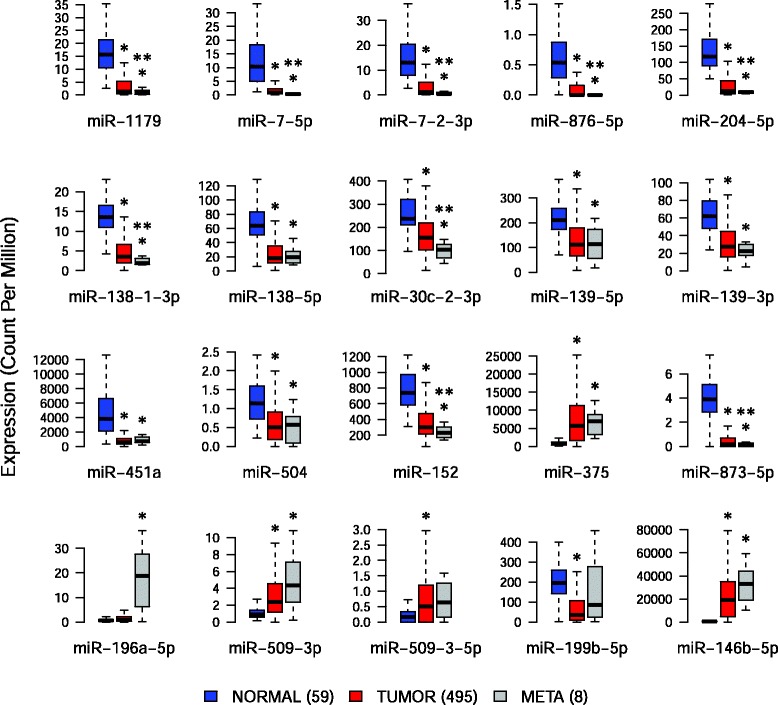
Validation of the differentially expressed selected microRNAs using the TCGA small RNA deep-sequencing data. Validation was performed on 59 normal samples, 495 primary tumors and 8 LNM. Unpaired *t*-test was used to compare the sample types. *: significant modulation (*p* < 0.05) of expression between normal samples and tumors or LNM. **: significant modulation of expression between tumor samples and LNM

We confirmed the down-regulations of the 14 microRNAs in tumors compared to normal samples (Fig. 5). We obtained the same results when we selected tumor samples based on a cell content filter (see Methods), which reduced the number of primary tumors to 120, and when we compared the 59 matched tumor and normal samples. Among these microRNAs, we confirmed the down-regulations obtained by qRT-PCR in LNM compared to primary tumors for miR-7-5p, miR-7-2-3p, miR-30c-2-3p and miR-152 but not for miR-139-5p. Additional down-regulated microRNAs between LNM and tumors were detected: miR-138-1-3p, miR-204-5p, miR-873-5p, miR-876-5p and miR-1179. Concerning miR-196a-5p, even if its expression is increased in LNM, its significant up-regulation between LNM and tumors observed by qRT-PCR was not validated in the TCGA data. No other modulations between LNM and tumors were found. The lack of confirmation of the differentially expressed microRNAs between primary tumors and LNM might result from differences in the cellular composition of the samples, and in particular from the presence of high amounts of immune cells in the LNM. We did not perform the analysis by comparing matched primary tumors and LNM or LNM samples selected on the basis of their high thyroid tumor cells content, because such selection resulted in a great loss of samples.

#### Down-regulated microRNAs can distinguish the two principal histological variants of PTC

**Fig. 6:**
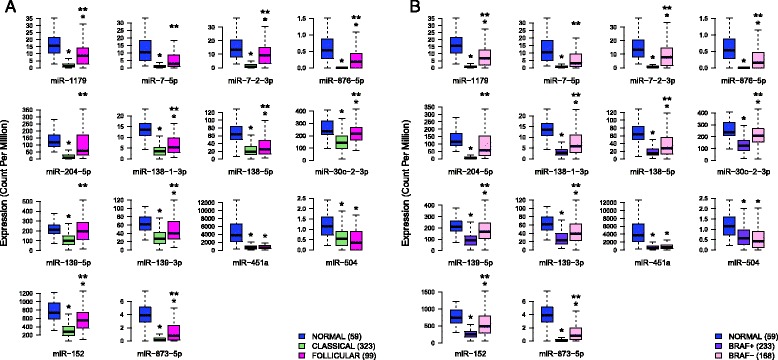
Down-regulated microRNAs in PTC are differentially expressed in tumor subtypes. A/B: the expressions of the 14 validated down-regulated microRNAs were investigated in in classical and follicular histological variants (**a**) and in BRAF V600E positive and negative tumor samples (**b**). For both, unpaired *t*-test was used to compare the sample types and subtypes. *: significant modulation (*p* < 0.05) of expression between normal samples and tumors. **: significant modulation of expression between tumor subtypes. Numbers in brackets correspond to the number of available samples in the TCGA dataset for each tissue type

To further analyze our results we compared the expression levels of the 14 down-regulated microRNAs between the normal samples (*n* = 59), the classical variants (*n* = 323) and the follicular variants of PTC (*n* = 99) in the TCGA dataset (Fig. 6a). Less frequent variants and non-annotated samples were not taken into account. 11 microRNAs are down-regulated in both variant types and, except for miR-451a and miR-504, they are also significantly less expressed in classical variants. miR-7-5p, miR-204-5p and miR-139-5p are only significantly down-regulated in the classical variants. These results suggest that, in addition to their ability to discriminate PTC from normal samples, the studied down-regulated microRNAs are also able to distinguish tumor histological major subtypes.

#### Down-regulated microRNAs can distinguish aggressive from non-aggressive phenotype of PTC

We obtained similar results when we compared the normal samples with the BRAF V600E positive PTC samples (*n* = 233) and the BRAF negative PTC samples (*n* = 168) in the TCGA dataset (Fig. 6b). Again, non-annotated samples were not taken into account. The same 11 microRNAs plus miR-139-5p are down-regulated in both tumor types but, except for miR-451a and miR-504, they are significantly less expressed in BRAF V600E positive samples. miR-7-5p and miR-204-5p are only significantly down-regulated in BRAF V600E positive tumors. These results were not unexpected taking into account that in the 191 BRAF V600E positive PTC samples annotated for variant type, 179 are classical and only 12 are follicular. In addition, in the 151 BRAF V600E negative samples annotated for variants, only 80 are classical and 71 are follicular. The difference of the histological variants repartition in the BRAF V600E negative and positive tumor populations was assessed by a two-side Fisher’s exact test which was very significant: pval < 0.0001.

Globally, similar results were obtained when comparing LNM positive N1 (*n* = 206) vs LNM negative N0 (*n* = 214) PTC samples and high risk (*n* = 24) vs low risk (*n* = 171) PTC samples (Additional file 1: figure S2). As expected, the proportion of BRAF mutated tumors in the aggressive population is significantly higher than in the non-aggressive population (Fisher’s exact test pval < 0.01 in both comparisons).

**Fig. 7:**
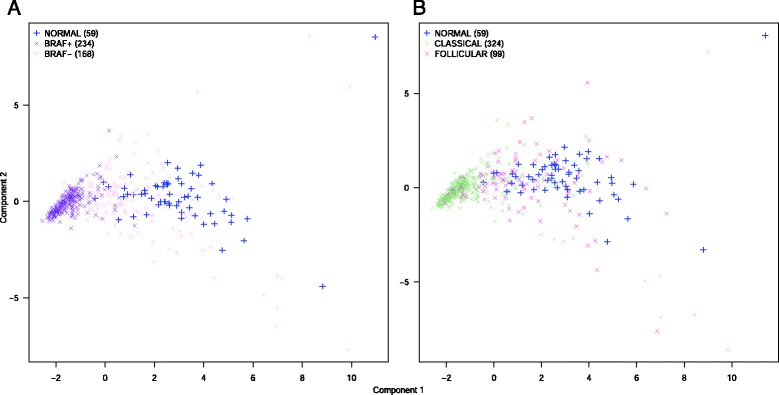
Principal component analyses of whole TCGA dataset. **a**/**b**: the analysis was performed on the 59 normal samples and annotated tumors for BRAF mutational status (**a**) or histological subtypes (**b**). Non annotated samples for one of these parameters were not used in the corresponding PCA. No additional filtering criterion was used. The analyses were performed with the expression of the 14 validated down-regulated microRNAs in PTC. Numbers in brackets correspond to the number of available samples in the TCGA dataset for each tissue type

In order to compare the discrimination ability of related clinical and pathological information, we performed different PCA analyses on the TCGA dataset with the expression of the 14 down-regulated microRNAs. Information regarding BRAF mutational status, histological subtype, clinical risk, presence of lymph node metastases, clinical tumor stage, presence of extra-thyroidal extension, age and gender were used to create different PCA plots. Non annotated samples for one of these parameters were not used in the corresponding plot. Normal samples were added to each PCA analysis (Additional file 1: figure S3). The BRAF V600E mutational status and the histological subtype variations showed the most effective separation between the analyzed samples (Fig. 7), suggesting that the variations of microRNA expressions between PTC samples are mainly explained by these parameters.

In conclusion, we have validated the modulation of expression of our microRNAs in independent samples. These down-regulated microRNAs are systematically less expressed in more aggressive PTC samples, in correlation with the presence of the BRAF V600E mutation.

### Deep investigation of the miRNome revealed no major implication of isoforms variation or arm shift in PTC tumorigenesis and lymph node metastatic progression

Since microRNA 5p-to-3p arm expression ratio modulations, NTA distribution and isomiRs distribution have been associated with tumorigenesis in several cancers [23–28], we investigated the presence of such variations in our deep-sequenced tumor and LNM samples (Table 1). We validated our results on independent samples from 14 patients by qRT-PCR when possible and with the TCGA dataset.

- ***▪ 5p-to-3p arm ratio***

We calculated for each sample type and each pre-miR the expression ratio between both microRNAs (5p arm versus 3p arm) encoded by the same pre-miR. This calculation was only performed for microRNAs precursors encoding both a 5p and 3p mature microRNA. Expression of mature arms was performed in two ways: (1) using all distinct isoforms identified for a given microRNA (2) using only its canonical isoform reported in miRBase (v19). We found 9 pre-miR for which the 5p arm versus 3p arm expression ratio showed a fold change above 1.5 or below 0.66 between sample types (let-7d, mir-17, mir-27b, mir-92a-1, mir-181a-1, mir-181c, mir-221, mir-324, mir-1307), but only one of them (pre-miR-17) had an inversion of the dominant arm. We performed Taqman and SYBR Green (Exiqon) qRT-PCR validation experiments for 3 selected pre-miR (mir-324, mir-17, mir-1307) on the deep-sequenced samples and the validation sample set. These two methods have been designed to target the canonical isoform reported in miRBase, unlike deep-sequencing. Although we confirmed the significant modulations observed for the 3 pre-miR in deep-sequenced samples, we only succeeded in validating the significant modulation observed for pre-miR-324 in the validation sample set. However, neither pre-miR-324 nor the 8 other pre-miR had a significant modulation of the 5p-to-3p arm expression ratio in the TCGA validation dataset with or without filtering out samples with low tumor cells content (Additional file 1: Figure S4). These data suggest that 5p-to-3p arm modulation is not a common feature of PTC tumorigenesis and lymph node metastatic progression.

- ***▪ NTA distribution***

**Fig. 8:**
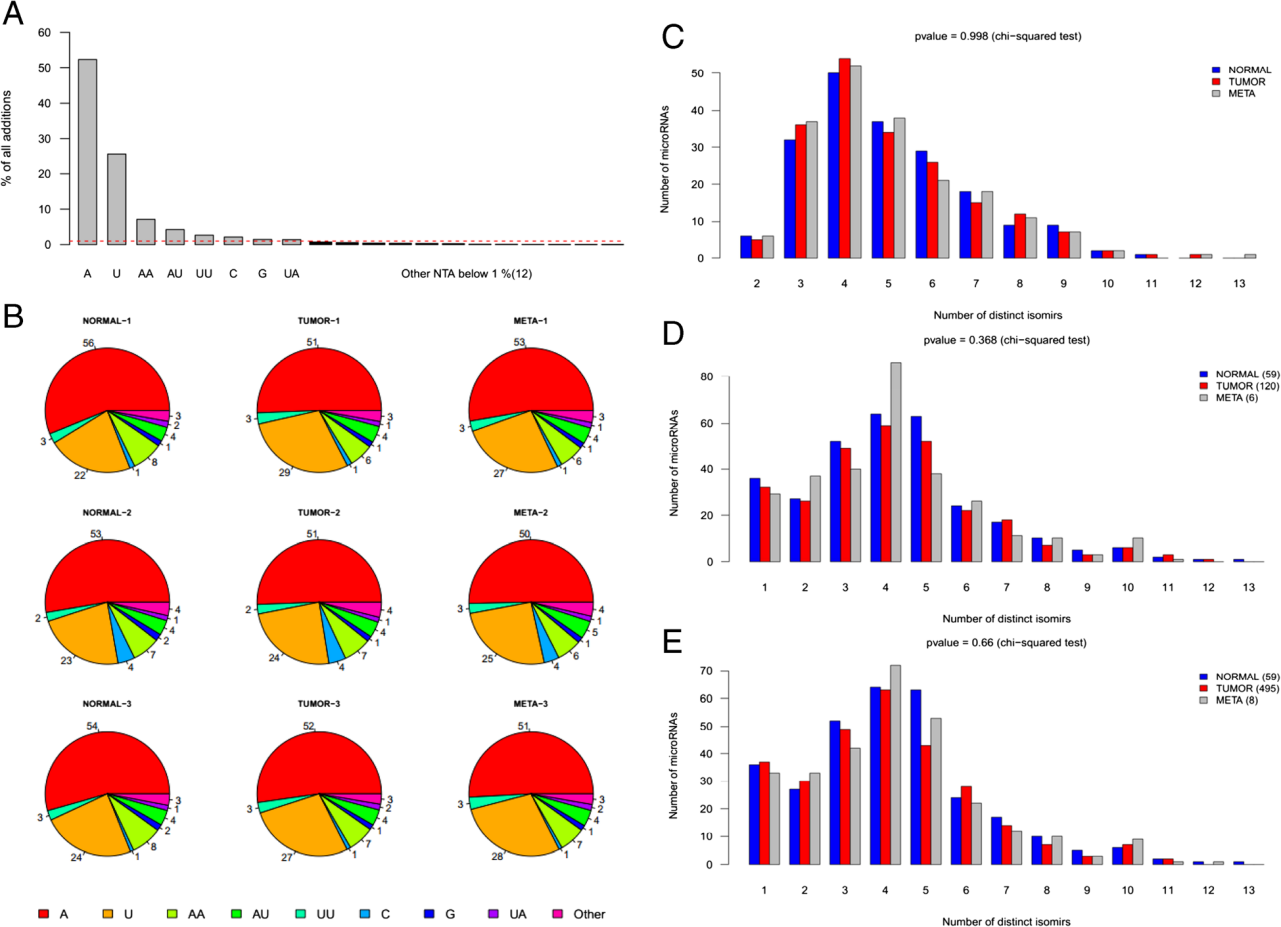
Three′ non-templated nucleotide additions and isomiRs distributions in PTC. **a**: the contribution of each individual addition was first computed for each mature microRNA in every of sample of the first sample set, and then averaged across all these samples. Normal samples, primary tumors and LNM from 3 patients were analyzed. Red dashed line corresponds to the minimum fraction of 1 % used to distinguish relevant additions from sequencing or trimming errors. **b**: only additions accounting for 1 % or more of all NTA in a given sample are represented. Other less frequent additions are grouped in the “other” category. Numbers correspond to the contribution, in percentage, of each type of addition averaged across all microRNAs having a minimum total mapped reads of 100. **c**/**d**/**e**: only isomiRs with a relative contribution to the total mapped reads of a mature microRNA of 1 % in at least one sample were considered. The number of isoforms per mature microRNA was then averaged by tissue type. The distributions were obtained in the 9 samples of the first sample set (**c**), in the TCGA samples dataset filtered based on clinical and pathological criteria (**d**) and in the complete TCGA dataset without additional filtering criteria (**e**). Numbers in brackets correspond to the number of available samples in the TCGA dataset for each tissue type. Eight available metastases samples were used. Independence of the distributions was assessed using a chi-squared test. The vast majority of microRNAs covered by a minimum of 100 reads have between 2 and 8 isomiRs

Non-templated additions were identified directly during read mapping. The pipeline associates to each tag the nucleotides that had to be removed at the 3′ end to obtain a match on the reference. First, we evaluated the contribution of each type of NTA across all microRNAs in all our deep-sequenced samples (Fig. 8a). As expected, the major contribution came from adenine and uracil nucleotides. Next, we represented the relative contribution of each type of NTA for every sample independently (Fig. 8b). In general, the contribution of each type of NTA is very similar across all samples. Then, we computed the fraction of adenylated and uridylated mapped reads for each mature microRNA in every samples. We identified the most adenylated and uridylated microRNAs in our samples and noticed that there was no overlap between them excepted for 2 microRNAs (Additional file 1: Figure S5). Furthermore, the majority of microRNAs had a level of adenylation or uridylation below 30 % of all mapped reads. We also screened NTA to identify specific microRNAs harboring different patterns of additions when comparing normal tissues to matching tumors and metastases, but we did not find variations of adenylation or uridylation accounting for at least 20 % of all mapped reads. This suggests that non-templated additions in human are tissue and microRNA specific, as previously suggested [37–40], but do not seem to be strongly related to tumor initiation or progression in the case of thyrocytes. Since the TCGA miRSeq public data only include per-sample isoform read counts and normalized expression, it was not possible to use them to reproduce this calculation (no information was available regarding the sequence of mapped reads).

- ***▪ isomiRs distribution***

We first compared the number of distinct isomiRs per mature microRNA between each of our deep-sequenced sample. A specific isomiR was considered present in a given sample if it accounted for at least 1 % of the expression of the mature microRNA. We found no difference between sample types. The vast majority of mature microRNAs, covered by a minimum of 100 reads presented between 2 and 8 isomiRs (Fig. 8c). We repeated this analysis with the TCGA data with or without filtering out samples with high tumor cells content but we found no difference between sample types (Fig. 8d,e). We then grouped isomiRs by classes, according to the modification of their start and end coordinates compared to their canonical counterpart. In order to analyze the distribution of these classes between sample types, we calculated the contribution of each class for every microRNA in the 9 samples that were deep-sequenced. We did not find any variation of these contributions between sample types (Additional file 1: Figure S6A). As expected, the canonical isoform defined in miRBase v19 was not always the most expressed. Indeed, we averaged class contribution across all 9 samples and the canonical isoform was the most expressed for only 48 % (93/194) of the selected microRNAs. The same values were observed for the end-site (3′) isomiR class. On the opposite, the start-site (5′) and both sites classes (5′and 3′) were dominant for only 1 % (2/194) and 3 % (6/194) of selected microRNAs respectively. We then redefined the canonical microRNA as the one having the highest relative contribution to total microRNA expression in the three normal samples. The classes were reassigned accordingly. In these conditions, the end-site (3′) class had still the most variable contribution across all selected microRNAs (ranging from 0 to 73 %) compared to classes involving 5’ coordinate shifts (ranging from 0 to 36 % and 0 to 30 % for start-site and both sites classes respectively) (Additional file 1: Figure S6B). Furthermore, there was still no variation of these contributions between sample types. We also searched for specific isomiRs whose contribution to total expression of the corresponding microRNA would be altered during the course of tumorigenesis and metastatic formation in the 9 samples. We found 5 microRNAs (let-7i-5p, miR-19b-3p, miR-27a-3p, miR-27b-3p, miR-148b-3p) for which at least one isomiR showed a variation of at least 20 % when comparing its mean contribution over normal tissues to primary tumors or LNM. However, all these isomiRs were 3′ variants of the canonical form, leaving the seed region unmodified. Additionally, we failed to confirm these observations using TCGA data. This suggests that isomiRs do not seem to be strongly related to tumor initiation or progression of thyrocytes.

### MicroRNA A-to-I editing is present in weakly expressed mature microRNA in thyrocytes

We designed a custom variant calling approach to deal with the extreme coverage of some highly expressed microRNAs. Read alignments of the 9 deep-sequenced samples confirmed the A-to-I edition of the miR-376 family, which was already reported in glioblastoma cells [26]. However, this microRNA is weakly expressed in each sample type. We identified new A-to-I edited positions. We identified 4 single nucleotide substitutions matching our filtering criteria but in weakly expressed microRNAs (Additional file 1: Table S3). In addition, editing frequencies obtained from read alignments did not show any significant difference between sample types. We further validated these editions by cDNA Sanger sequencing with primers designed to target the pri-miR sequence (Additional file 1: Table S4), and confirmed the absence of substitution in the DNA of these samples by genomic DNA sequencing. A-to-I RNA-editing (recognized as a G by the sequencer) was found for miR-605-3p in each sample type (Additional file 1: Figure S7). We also screened read alignments for other mutations located in mature microRNAs and potential variation of the mutational status between sample types but we did not find any candidate mutation.

## Discussion

In our study, we searched for new microRNA biomarkers of PTC tumorigenesis and lymph node metastasis formation. We used a custom bioinformatics framework for the analysis of the microRNA expression profiles of 3 PTC, matched normal samples and lymph node metastases. The choice of a first limited but well characterized set of samples is completely assumed. Indeed, this could lead to a loss of non-common variations in each sample but in this study we looked for strong and common new biomarkers of PTC. These samples were carefully selected on the basis of their normal or tumor cells composition to avoid cell contamination which would bias the results [41–43]. These samples were used as a small “training” set to find modulations in the whole miRNome, which were then validated in a very large amount of samples by two different approaches. First, by qRT-PCR on independent samples from 14 other patients. These samples were also carefully selected based on their normal or tumor cell composition. Secondly, we validated the results by an *in silico* analysis of the TCGA database which is the largest available dataset of PTC to our knowledge [30]. qRT-PCR and *in silico* data were not always concordant. This can be due to different reasons. First, the number of samples analyzed is quite different between the 2 datasets. This variation changes the power of the associated statistics. Secondly, the methodologies are conceptually different [21]. Finally, unlike qRT-PCR dataset, the TCGA dataset does not allow to adjust for interindividual variations by patient matched analyses without a great loss of information regarding LNM samples and not all the samples have been selected on the basis of their cell composition.

Our study revealed the strong ability of down-regulated microRNAs in PTC to distinguish tumors and LNM from normal thyroid samples. Their specific expression in thyrocytes was confirmed experimentally by LCM analyses, and then validated experimentally and *in silico* by selecting tumors with a low content of contaminating cells. Unlike commonly reported up-regulated microRNAs in PTC, very few down-regulated microRNAs were identified in microarrays studies [8, 20]. Only recent small RNA deep-sequencing studies showed a common list of down-regulated microRNAs in PTC [27, 30, 44]. However, their functions and abilities to distinguish sample types and tumor subtypes were little explored. This is the reason why we decided to focus our study on these microRNAs. In 2013, Swierniak et al. reported a list of 44 up- and down-regulated microRNAs in 14 PTC samples compared to matched normal thyroid samples [27]. They used small RNA deep-sequencing analysis and validated their results by microarray on 9 additional sample pairs. Recently, Mancikova et al. described a smaller list composed by 10 up-regulated and 5 down-regulated microRNAs in PTC compared to normal samples [44], by performing small RNA deep-sequencing analysis on a training set of 35 PTC and 8 normal samples and a validation set of 43 PTC and 9 normal samples. Several microRNAs were common between both studies, but there were also differences. The number of samples considered, the RNA extraction method used and the bioinformatics pipeline used for the analysis of the deep-sequencing data may explain the discrepancies between the different studies. In our study, we identified by small RNA deep-sequencing analysis 31 up-regulated and 30 down-regulated microRNAs in PTC (classical variants) compared to matched normal samples. We confirmed that, as already described for up-regulation, consistent down-regulation of microRNAs occurs as well during PTC tumorigenesis. The list of the 14 most down-regulated microRNAs that we validated by qRT-PCR and *in silico analyses,* on subtypes of PTC, extends the observations of Swierniak et al. on the largest available set of PTC samples. In addition, we have extended these observations to LNM and have shown that these selected down-regulated microRNAs are differentially expressed between the 2 major histological subtypes of PTC (classical and follicular). Among the described down-regulated microRNAs, miR-7-5p has already been studied as a diagnostic marker for PTC but with limited efficacy (sensibility 82 %, specificity 73 %) [8, 45]. Accordingly in our work, miR-7-5p was not significantly modulated in follicular variants of PTC compared to normal samples. However, since it has been shown that a diagnostic signature based on multiple microRNA expressions can outperform previous single microRNA signatures [8], we propose to combine the down-regulated microRNA described in this study to the panel of microRNAs already explored to define a very efficient diagnostic signature of PTC in thyroid biopsies. However, more studies are required to assert the efficiency of any microRNA signature on rare subtypes of PTC, like diffuse sclerosing variants.

Many publications report that PTC samples with different degrees of aggressiveness present different microRNA expression profiles [46–48], on the basis of microarrays and qRT-PCR analyses. These studies revealed the common up-regulation of the well-characterized miR-146b-5p, miR-221-3p and miR-222-3p in aggressive PTC compared to non-aggressive samples but no commonly down-regulated microRNAs. Since the BRAF V600E mutation has been associated with PTC aggressiveness [49–51], Swierniak et al. searched for a BRAF V600E associated expression profile in their small RNA deep-sequencing data but without success [44]. However, the recent global analysis of the TCGA data revealed that the BRAF mutational status deeply modulate the wide transcriptome of PTC [30]. In particular miR-7-5p, miR-204-5p and other microRNAs expressions in PTC are associated with the presence of the BRAF V600E mutation. In our study, we found that 12 of the 14 strongest down-regulated microRNAs in PTC were significantly less expressed in BRAF V600E positive PTC samples compared to negative PTC samples. In addition, miR-7-5p and miR-204-5p were not significantly down-regulated in BRAF V600E negative PTC samples compared to normal samples. In accordance with our observations, Mancikova et al. recently showed that miR-7-5p and miR-204-5p expressions are deeply associated with the BRAF V600E mutational status [44]. We found similar results when we compared tumors with a “high” clinical risk to tumors with a “low” clinical risk and tumors with LNM (N1) to tumors without LNM (N0), suggesting the general implication of the down-regulated microRNAs in the aggressiveness of the tumor. The “aggressiveness” of a tumor is a complex notion which involves clinical and pathological aspects like BRAF V600E mutational status, histological subtype, clinical tumor stage… We showed that the variations of microRNA expressions between PTC sample subtypes seemed mainly related to the BRAF V600E mutational status. This suggests that the constitutive activation of the RAS/RAF/MAPK pathway by the BRAF V600E mutation results in a higher deregulation of microRNA expressions. However, it is not clear if these modulations are a cause or a consequence of the aggressive phenotype.

The majority of the down-regulated microRNAs have been reported to be “tumor suppressor” microRNAs in other cancers. This is the case for miR-7-5p [52, 53] and miR-204-5p. Interestingly, one of the validated targets of miR-7-5p is EGFR (epidermal growth factor receptor) mRNA [54, 55] which is a major inducer of thyrocyte proliferation. miR-204-5p has been reported as a repressor of both MMP3 and MMP9 (matrix metalloproteinase) mRNA and also as a direct repressor of HMGA2 (High-mobility group AT-hook 2) mRNA. The proteins encoded by these mRNAs play an active role in the mobility and the invasiveness of cancer cells [56, 57]. So, in addition to their role as potential strong biomarkers candidates these microRNAs probably play a functional role in tumor progression.

The implication of microRNAs in lymph node metastasis formation is only poorly characterized. It has been shown in different cancer types that microRNA expressions in LNM reflect the microRNA expressions in the associated primary tumors [58–60]. Some studies suggest that LNM formation does not require major microRNA modulations [60] whereas another reports differentially expressed microRNAs between primary tumors and associated LNM [58], suggesting an implication of microRNAs in LNM formation. Concerning PTC metastasis development, very little is known. Our small RNA deep-sequencing results revealed similar microRNA expression profiles between primary tumors and associated LNM. In addition, we failed to validate by qRT-PCR 6 of the 7 selected microRNA modulations between tumors and LNM and only 2 (miR-873-5p, miR-876-5p) were validated in the TCGA dataset. However, among the validated down-regulated microRNAs in PTC, some of them are nevertheless significantly less expressed in the LNM than in the primary tumors, such as miR-7-2-3p, miR-30c-2-3p or miR-873-5p. The number of microRNA varied between our first deep-sequencing analysis, qRT-PCR and *in silico* validations but increased with the number of samples considered. We also showed that miR-196a-5p is up-regulated in LNM containing at least 70 % of tumor cells. In other cancers, miR-196a-5p and miR-30c-2-3p have also been respectively identified as up- [61–63] and down- [64, 65] regulated during migration and invasion. So, although the microRNA expression profiles of primary tumors of PTC and LNM are very similar, some microRNAs are modulated and could participate to metastasis formation. However, the amount of LNM used in our study is limited and further studies are therefore required to confirm these preliminary data as well as to characterize the function of these microARNs in LNM formation.

Our results showed that there is no major change in the 5p-to-3p expression ratios, the isomiR distribution and the NTA distribution which could be involved in PTC tumor development and constitute new biomarkers. However, our analyses are limited by the small number of samples in our “training” set. Furthermore since the TCGA small RNA deep-sequencing public data only include per-sample isoform read counts and normalized expression, it was not possible to use them to validate the results of the NTA analysis. In addition, unlike the TCGA mapping strategy, our read mapping strategy was designed to specifically distinguish 3′ NTA from isomiRs. It is thus possible that a given isoform “A” reported in the TCGA data actually corresponds to isoform “B” with one or several non-templated additions in our own dataset. This limits the accuracy of comparisons of isomiR distribution. Nonetheless, we showed that the large majority of microRNAs had a level of adenylation or uridylation below 30 % of all mapped reads and it is rare to find microRNA with a high level of adenylation and uridylation at the same time. This is in accordance with previous results [37, 66]. This particular issue only concerned isomiRs characterization and had no effect on expression profiles, obtained using all isoforms identified for a given mature microRNA. Additionally, qRT-PCR is inefficient for the validation of the results of the NTA and the isomiR analyses.

However, to our knowledge, this is the first work which explores 5p-to-3p expression ratios and NTA distribution in PTC. IsomiR distribution has already been studied by Swierniak et al. who showed that this distribution changes during PTC tumorigenesis [27]. In accordance with them, we showed that the vast majority of mature microRNAs, covered by a minimum of 100 reads, presented between 2 and 8 isomiRs. However as opposed to what they report, we did not find modulation of the distribution between sample types, even when considering all the samples from the TCGA dataset. This discordance could be explained by the number of samples considered and by technical differences. Indeed, Swierniak *et al* used normal samples, normal tissues adjacent to tumor samples and tumors from 14 patients. In addition, they required perfect matches without any 3’ end NTA trimming when aligning reads. The selection of isomiR is also slightly different. We therefore concluded that the distribution of well-expressed isomiRs is similar in normal thyroid samples and PTC.

This absence of major modulation contradicts several studies on different cancer types which have mentioned that 5p-to-3p expression ratios, NTA and isomiR distributions may be altered in tumors [23–25, 27, 28]. Two non-exclusive hypotheses may explain these discrepancies: (1) there is no such modulation in the thyrocytes, neither during tumorigenesis nor during LNM development; (2) these modulations are more related to cellular contamination and read mapping strategy than to tumorigenesis. Studies reporting such modulations involve tumor samples without well-defined cellular content, and the mapping strategy differs between studies [23–25, 27, 28]. Indeed, contaminating cells like lymphocytes or fibroblasts and even normal cells could alter expression profiles and decrease the specific signal of the tumor cells [41–43]. However, even when using the full set of unfiltered TCGA samples, no modulation of 5p-to-3p expression ratios or isomiR distribution was found between the sample types.

microRNA editing was observed in thyrocytes, and we were able to validate a A-to-I editing event occurring in miR-605-3p. As the edited base was located in the seed sequence, this specific editing event may change the mRNA targets of this microRNA. However, no modulation was observed between samples types. We concluded that editing may be indeed present in mature low-expressed microRNAs in thyrocytes but does not seem to contribute to PTC tumorigenesis.

In our study, we developed a new flexible bioinformatics framework to explore every aspect of the miRNome. Small RNA deep-sequencing outperforms traditional quantitative technologies like qRT-PCR or microarrays [21]. However, the absence of “gold standard” read mapping guidelines decreases the comparability of the studies and increases the risk of technical biases in the results. In 2010, De Hoon et al. [29] showed that, with an inappropriate analysis pipeline, a microRNA read may be mapped on a wrong location in the reference genome. This effect, known as cross-mapping effect, may dramatically change the conclusion of the analysis by creating wrong sequence variations and microRNA quantifications. Two years later, Alon et al. [22] showed that an alignment of the microRNA reads against the whole genome with up to one mismatch tolerated and a 3′ end NTA trimming procedure drastically decreases the level of cross-mapping. Recently, Muller at al. [66] showed that an analysis pipeline which does not consider isomiRs and NTA variations may result in a loss of 31 % of the microRNA expression signal. Indeed without isoform detection the number of individual microRNAs presenting more than 100 reads in at least one sample of the study decreased from 318 to 219 microRNAs. In our study, we designed our bioinformatics analysis pipeline in order to meet guidelines established by the previously mentioned authors: (1) the microRNA reads were aligned directly to the whole genome, because small RNA libraries also contain other short RNAs (snRNAs, tRNAs…) which could be confounded with mature microRNAs having a similar sequence; (2) the isomiR variations were captured by considering all reads mapped within a 5 base pairs window of every canonical microRNA; (3) the 3′ end NTA were iteratively trimmed until a match could be found and the information regarding the trimmed nucleotides were kept; (4) the number of allowed mismatches was gradually increased by a multistep alignment procedure so that perfect matches were always preferred. With this strategy, up to 93 % of individual reads could be mapped during the first step when using the human genome hg19 as reference (Fig. 1 and Methods). Considering that no “gold standard” exists in the literature and that all the authors do not explain in details their bioinformatics framework in such a way that non experts can reproduce the analyses, we addressed alignment issues previously reported in the literature and proposed unified guidelines to study every aspect of microRNA biogenesis from each small RNA deep-sequencing data in an unbiased way.

## Conclusions

Down-regulation of microRNAs is a common feature occurring during PTC tumorigenesis. This observation was expanded to lymph node metastases and we showed that these modulations occur specifically in the thyroid tumor cells. These modulations are associated with the aggressiveness of the primary tumor and are further amplified in BRAF V600E positive cells. Our results suggest that the observed down-regulated microRNAs in PTC are not only potential strong biomarker candidates but may have a functional role at different levels during thyroid tumorigenesis and metastases formation. This should be explored by functional studies.

The first complete miRNome analysis of PTC that we performed showed that there is no major change in the 5p-to-3p expression ratios, the isomiRs and the NTA distributions, or in the A-to-I editing sites of mature microRNAs, suggesting that they do not contribute to PTC tumor development.

Considering that no “gold standard” exists in the literature, we propose unified guidelines to analyze each aspect of microRNA deep-sequencing data in an unbiased way.

### Availability of supporting data

The data sets supporting the results of this article are available in the NCBI’s Gene Expression Omnibus repository, [http://www.ncbi.nlm.nih.gov/geo/query/acc.cgi?acc = GSE57780] and from Broad Institute’s Genome Data Analysis Center, [https://confluence.broadinstitute.org/display/GDAC/Download].

## Additional files

Additional file 1:
**Figure S1 to S9 and Table S1 to S4.** (PDF 1467 kb) Additional file 2:
**Supplemental materials and methods.** (PDF 652 kb)
