# Supplementary material for: New global analysis of the microRNA transcriptome of primary tumors and lymph node metastases of papillary thyroid cancer
Source: BMC Genomics. 2015 Oct 21;16:828. doi: 10.1186/s12864-015-2082-3 (PMC4618137; doi:10.1186/s12864-015-2082-3)
Supplement: Additional file 1: — Figure S1 to S9 and Table S1 to S4. (PDF 1467 kb) [file 12864_2015_2082_MOESM1_ESM.pdf]

Supplementary figures and tables

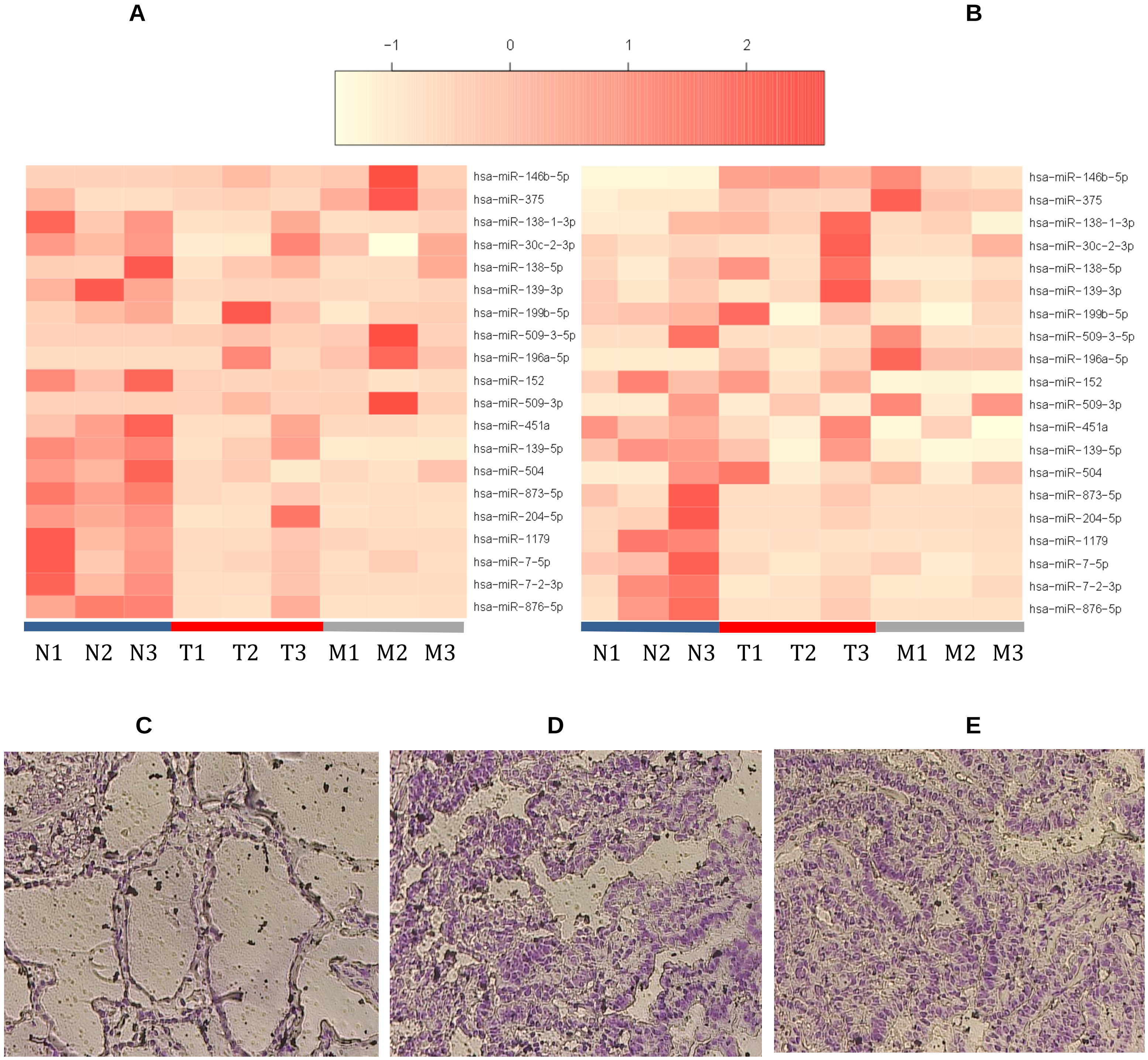

Figure S1. Quantitative RT-PCR validations on small RNA deep-sequenced samples. A/B: relative quantification was calculated with the Pfaffl method and normalized between samples based on U6 SnRNA expression. (**A**) heatmap of the expressions of the 20 microRNAs measured by qRT-PCR in each deep-sequenced. A significant correlation between fold changes (tumor/normal or metastasis/tumor) obtained for these microRNAs by deep-sequencing and by qRT-PCR (Spearman r= 0.64, P=0.0023) was found. Normal samples are underscored in blue (N), tumors (T) in red and LNM in gray (M). Expression

levels were scaled independently for each microRNA. (**B**) heatmap of the expressions of the 20 microRNAS measured by qRT-PCR in each microdissection from the deep-sequenced samples. A significant correlation between fold changes (tumor/normal or metastasis/tumor) obtained by qRT-PCR for these microRNAs on deep-sequenced samples and on the microdissections of the same samples (Spearman r= 0.63, P=0.0045) was found. **C/D/E**: cresyl violet staining of the microdissections is provided for a sample of normal tissue (**C**), tumor (**D**) and LNM (**E**).

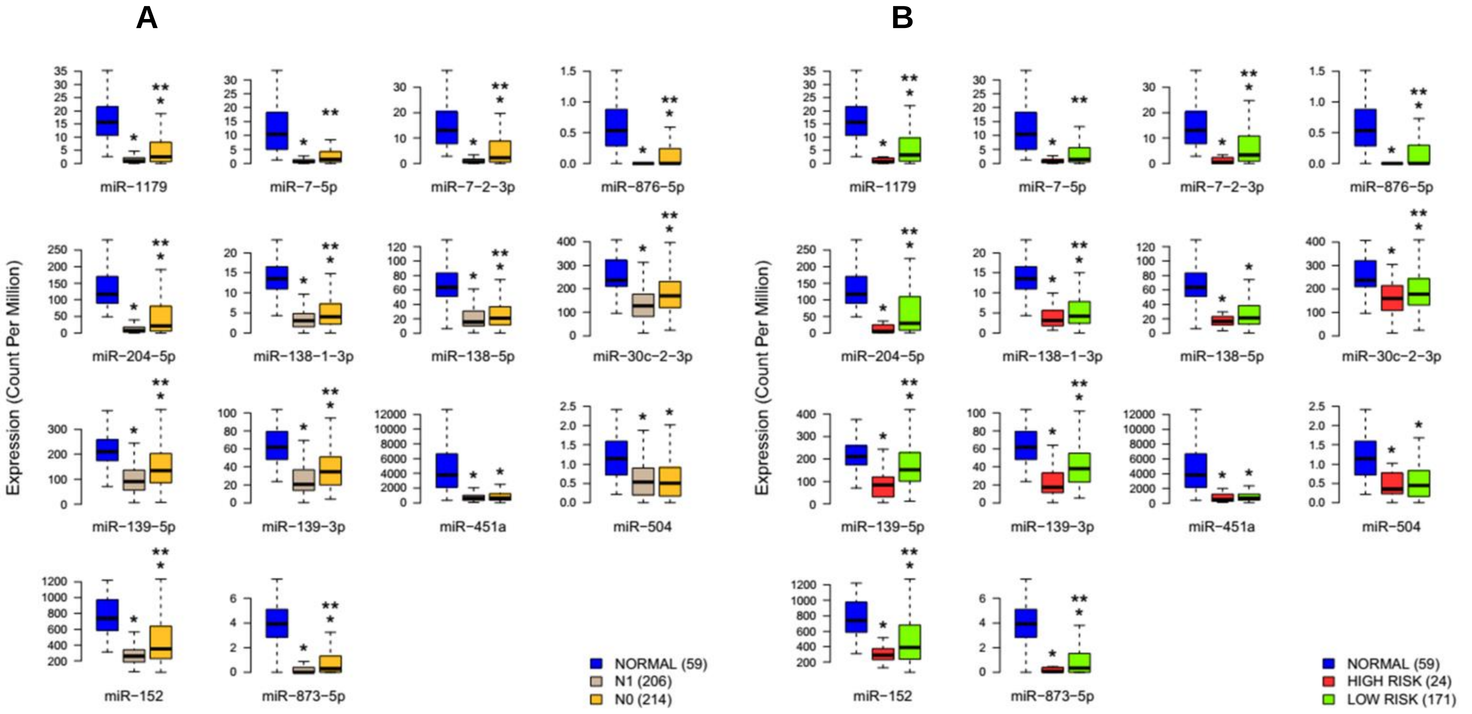

Figure S2. Down-regulated microRNAs in PTC are differentially expressed in tumor subtypes, supplemental results. A/B: the expression of the 14 validated down-regulated microRNAs was investigated in LNM positive (N1) and negative (N0) primary tumors (**A**) and in high risk and low risk tumors as defined clinically (**B**). For both, we used unpaired t-test to compare the sample types and subtypes. *: significant modulation ($p<0.05$) of expression between normal samples and tumors. **: significant modulation of expression between tumor subtypes. Numbers in brackets correspond to the number of available samples in the TCGA dataset for each tissue type.

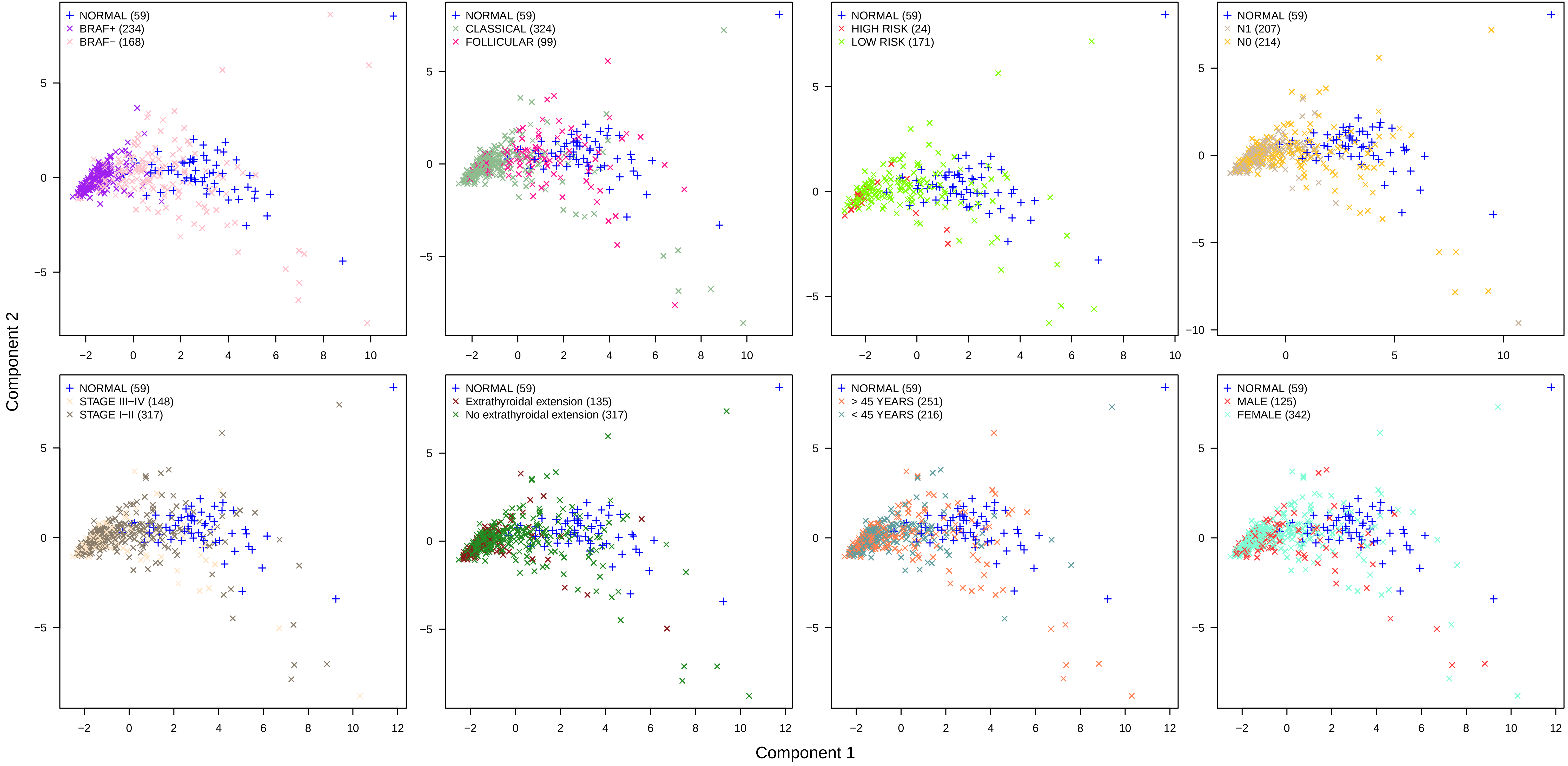
NORMAL (59)
BRAF+ (234)
BRAF− (168)
NORMAL (59)
CLASSICAL (324)
FOLLICULAR (99)
NORMAL (59)
HIGH RISK (24)
LOW RISK (171)
NORMAL (59)
N1 (207)
N0 (214)
NORMAL (59)
STAGE III−IV (148)
STAGE I−II (317)
NORMAL (59)
Extrathyroidal extension (135)
No extrathyroidal extension (317)
NORMAL (59)
> 45 YEARS (251)
< 45 YEARS (216)
NORMAL (59)
MALE (125)
FEMALE (342)
Component 1
Component 2

Figure S3. Principal component analyses of the whole TCGA dataset, supplemental results. The analysis was performed on the 59 normal samples and annotated tumors for different parameters of tumor aggressiveness: BRAF mutational status, histological subtype, clinical risk, presence of lymph node metastasis, clinical tumor stage, presence of extra-thyroidal extension, age and gender. Non annotated samples for one of these parameters were not used in the corresponding PCA. No additional filtering criterion was used. The analyses were performed with the expression of the 14 validated down-regulated microRNAs in PTC. Numbers in brackets correspond to the number of available samples in the TCGA dataset for each tissue type.

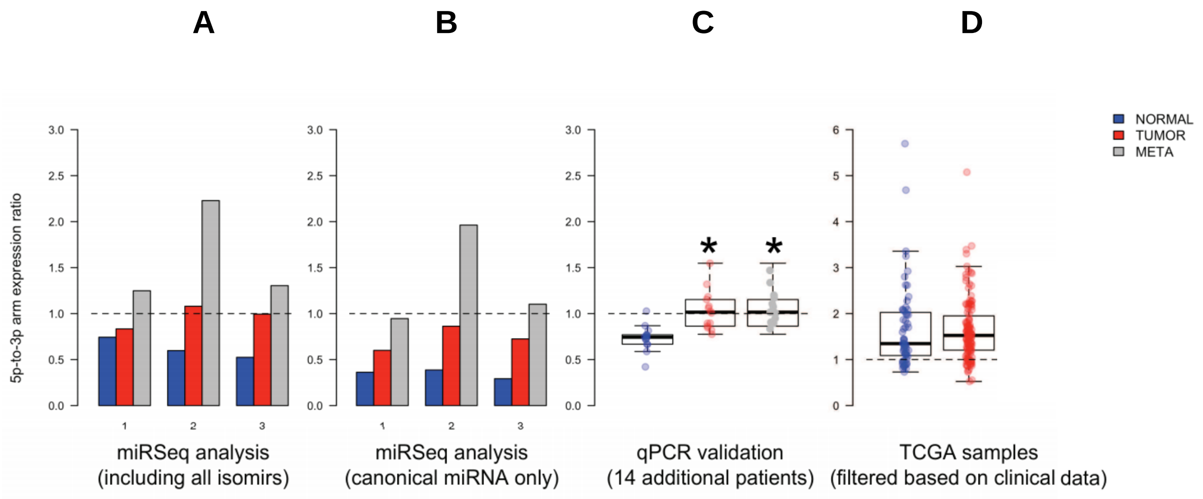

Figure S4. Evolution of the 5p/3p arm expression ratio of miR-324 between sample types. A/B: analysis was performed on our deep-sequencing results. **C:** only results from the qRT-PCR TaqMan assays are represented but ExiLENT SYBR Green assays showed similar results. We used paired t-test to compare sample types in the qRT-PCR results. *: significant modulation ($p<0.05$) of expression between normal samples and tumors or LNM. Absolute quantification was calculated based on dilution curves. For each sample, values obtained for each mature microRNAs arm were converted to 5p/3p ratios. **D**: this validation was also performed on 59 normal samples and 120 tumors matching our filtering clinical and pathological criteria from the TCGA small RNA deep-sequencing dataset. For each sample, values obtained in CPM corresponding to each mature microRNAs arm were converted to 5p/3p ratios but no significant variation of this ratio was found between sample types.

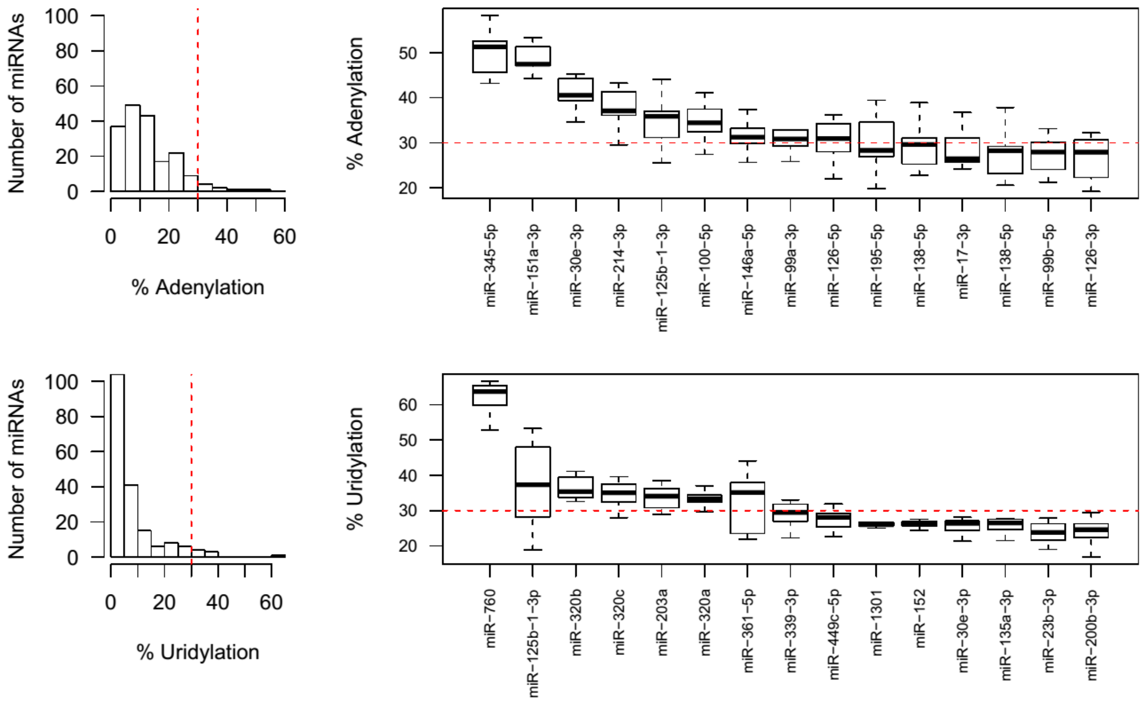

Figure S5. Pattern of adenylation (top) and uridylation (bottom) in the first sample set. The contribution of each individual addition was first computed for each mature microRNA (Total Mapped Read>100) in each sample, and then averaged across all samples. 3 normal samples, 3 primary tumors and 3 LNM from 3 different patients were used. The red dashed line represents 30 % of adenylated or uridylated reads per mature microRNA. More microRNAs undergo adenylation (top left) compared to uridylated microRNAs (bottom left). Top adenylated (top right) and uridylated (bottom right) microRNAs do not show any overlap except for miR-125b-3p and miR-30e-3p.

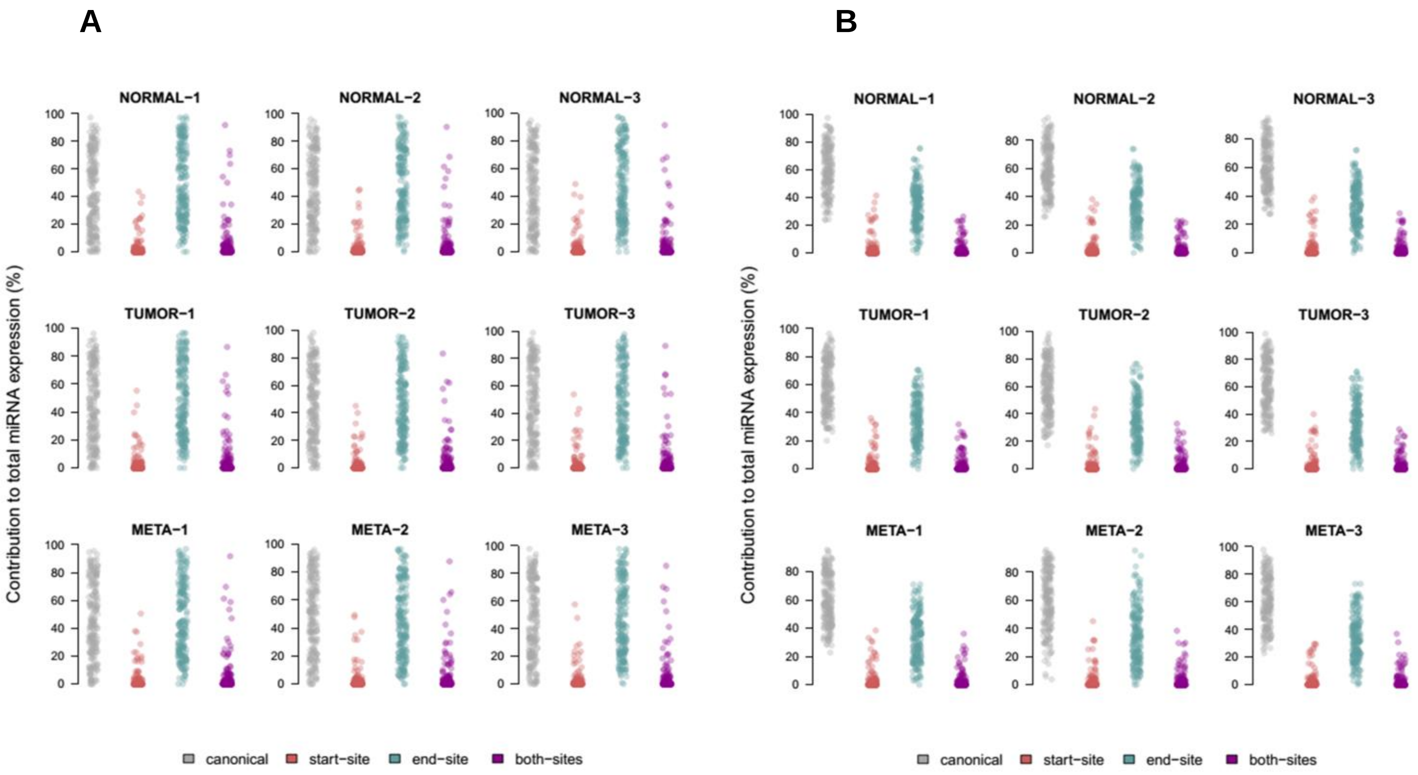

Figure S6. Contribution of each class of isomiR to the global microRNAs expression per samples in the first sample set. A: classes are defined relatively to the reference mature microRNA from miRBase v19. Each mature microRNA is defined by 4 dots, corresponding respectively to the relative contribution of each class of isomiR to its total expression. **B**: canonical microRNA is chosen based on the most abundant isomiR in all three normal tissues, and classes are reassigned accordingly. In both cases, end-site class has the most variable contribution compared to classes involving 5' coordinate modifications**.**

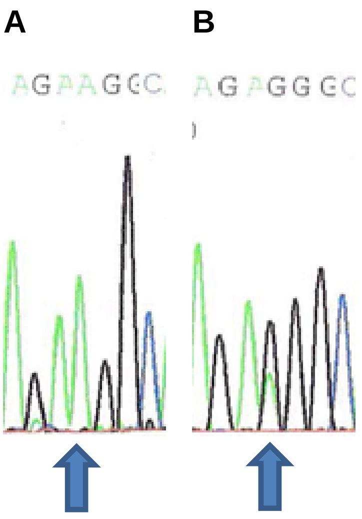

Figure S7. A-to-I RNA-editing of miR-605-5p in the first sample set. A: genomic DNA sequencing of miR-605-3p, the arrow shows the position of the expected edition. No mutation was found at this position. **B**: cDNA sequencing of miR-605-3p, the arrow shows the position of the A to I (G) RNA-editing. This was confirmed in each sample analyzed (normal, tumor or LNM).

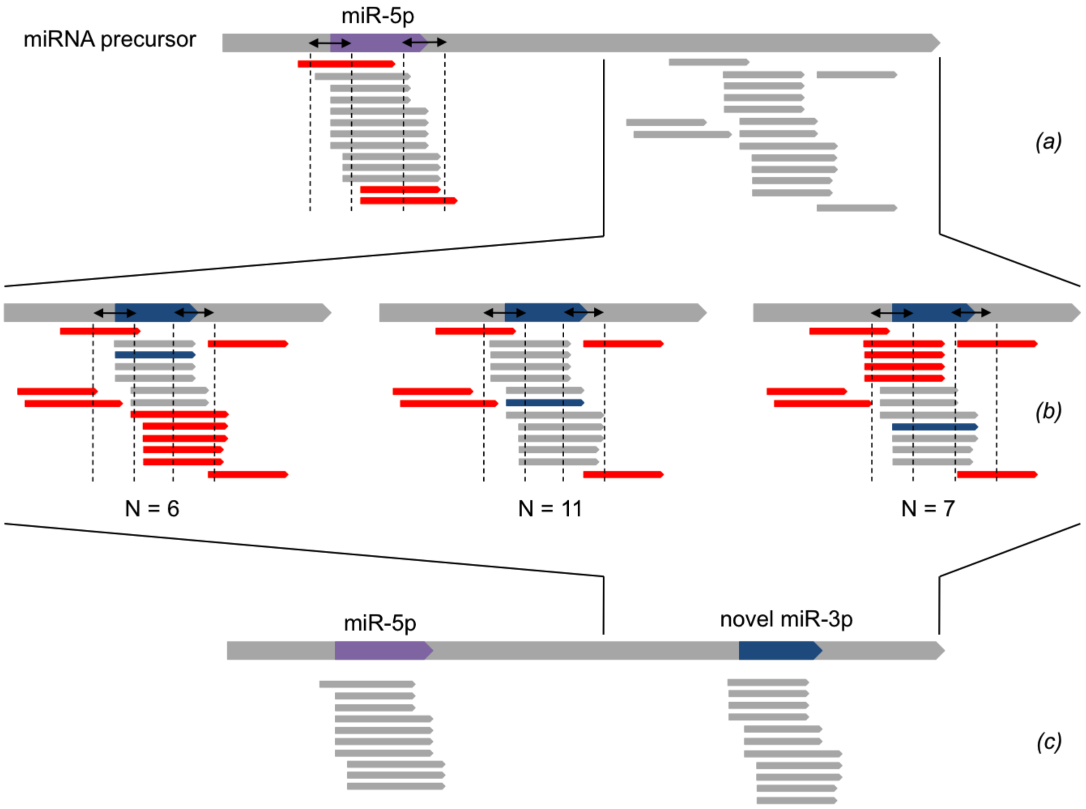

Figure S8. Identification of unknown 5p or 3p microRNAs in small RNA deep sequencing data. a: reads mapped on known pre-microRNA locations are collected across all libraries. Reads overlapping annotated microRNAs are removed (red) if their alignment coordinates are not located within a maximum distance of 5 bp compared to their respective canonical microRNA. B: reads that do not overlap annotated microRNAs are processed iteratively to determine the number N of individual reads that would cluster with them with respect to the specified windows. Reads that do not fit within the specified windows are colored in red, while the putative canonical microRNA is colored in blue. C: if the putative new canonical microRNA which clustered with the highest number of individual reads (N=11) meets the required depth and quality criteria, it is labeled as novel microRNA. Random matches are removed from alignments prior differential expression analysis.

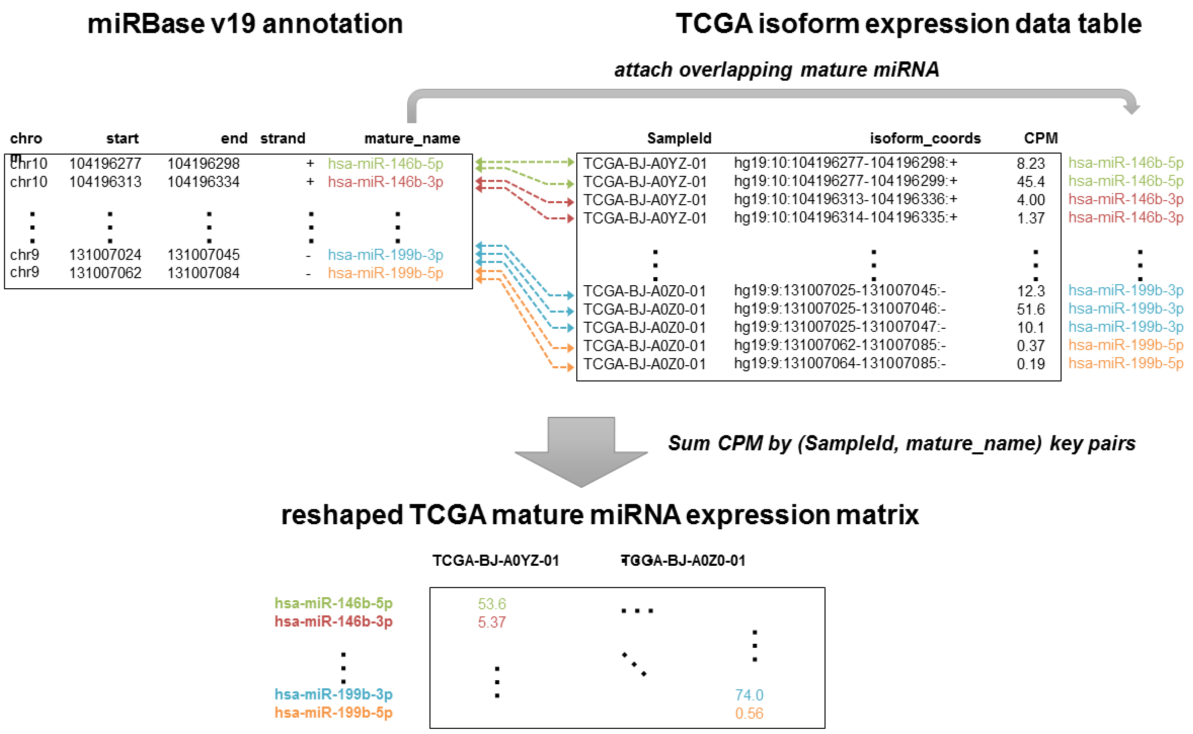

Figure S9. Processing of TCGA data tables applied to obtain per-sample mature microRNA expression. Each isoform listed in the TCGA data table is first attached to its overlapping mature microRNA with respect to miRBase V19 annotation. Mature microRNA expression levels are further obtained by summing CPM values by sample-miR pairs. Note that for better understanding, some fields originally present in the downloaded TCGA data table were omitted (e.g. *read_count, microRNA_region*) and the *reads_per_million_microRNA_mapped* field was renamed as CPM in the figure. Also, TCGA barcodes (*SampleId*) were truncated to contain only participant IDs and sample types.

Patient ID	Gender	Age (years)*	Diagnosis**	TNM	BRAF V600E
1	F	25	PTC	pT2 N1b M0	positive
2	M	73	PTC	PT4 N1b M0	positive
3	F	26	PTC	pT1 N1b M0	negative
4	F	45	PTC fv	pT3 N1b M0	negative
5	F	49	PTC	pT3 N1b M0	positive
6	F	26	PTC	pT3 N1a M0	positive
7	F	63	PTC	pT3 N1b M0	positive
8	F	10	PTC dsv	pT3 N1b M0	negative
9	F	24	PTC	pT3 N1b M0	negative
10	F	36	PTC	pT3 N1b M0	positive
11	M	39	PTC	pT2b N1a M0	negative
12	F	36	PTC fv	pT3 N1b M0	negative
13	F	33	PTC	pT3 N1b M0	positive
14	F	33	PTC	pT3 N1a M0	positive
15	F	41	PTC	pT1b N1b M0	negative
16	F	39	PTC fv	pT3 N1b M0	negative
17	M	57	PTC	pT3 N1b M0	positive

Table S1. Clinical and pathological information of the samples. Samples from patient 1 to 3 were used for the small RNA deep-sequencing and the qRT-PCR confirmation experiments, samples from patient 4 to 17 were used for the qRT-PCR validation experiments.* Age at the time of operation. ** PTC: classical variant of PTC; PTC fv: follicular variant of PTC; PTC dsv: diffuse sclerosing variant of PTC.

hsa-miR-135a-3p
hsa-miR-1180-5p
hsa-miR-181b-3p
hsa-miR-511-3p
hsa-miR-5699-5p
hsa-miR-133a-5p
hsa-miR-887-5p
hsa-miR-328-5p
hsa-miR-210-5p
hsa-miR-653-3p
hsa-miR-504-3p
hsa-miR-3909-5p
hsa-miR-450a-3p
hsa-miR-511-3p
hsa-miR-605-3p
hsa-miR-874-5p
hsa-miR-599-5p
hsa-miR-133a-5p

Table S2. List of microRNAs expressed in the deep-sequenced sample set that were not described in miRBase v 19.

microRNA	sequence	Coverage (reads)
hsa-miR-3679-5p	UGAGGAUAUGGCAGGGAAGGGGA	27
hsa-miR-605-3p	AGAAGGCACUAUGAGAUUUAGA	10
hsa-miR-6503-3p	GGGACUAGGAUGCAGACCUCC	198
hsa-miR-1260b	AUCCCACCACUGCCACCAU	12

Table S3. Sequences of potentially A-to-I microRNA-editing in the deep-sequenced samples. The edited nucleotide is labeled in red. The coverage is the mean of the number of reads mapped in each sample.

Genomic DNA

Targeted microRNA	Forward primer (5'->3')	Reverse primer (5'->3')
hsa-miR-3679-5p	TCTGTTTTCCTTTGGCACCT	GGGAAGGCAATCAAGATGAA
hsa-miR-605-3p	CGCCTCTTTTTGCTCATTCT	GTCATTGAGAGGCCAGGAAA
hsa-miR-6503-3p	GGAAGATGAAAGTGCTTTAAAATG	CCAGGAAAATTCTCTCATTCCA
hsa-miR-1260b	CAGGTGACATCTTGGCCTTT	GCTCCTGAGCCTTGTACAGC

cDNA

Targeted microRNA or gene	Forward primer (5'->3')	Reverse primer (5'->3')
hsa-miR-3679-5p	CTTCGTCCCCTTTCCTGTAA	GGACACCGCATGATGAAGAT
hsa-miR-605-3p	CGCCTCTTTTTGCTCATTCT	AGGTAACCTGTATCTGCAGTCCT
hsa-miR-6503-3p	TTCAGCTGCAAATTTAATCCTTT	CCATATCTTCGTGATTGTAAGCAG
hsa-miR-1260b	CAATCAGGGCTGAGTTCCTC	CTCCCAAGCAGCAGCAAC
BRAF	GCACAGGGCATGGATTACTT	GATGACTTCTGGTGCCATCC

Table S4. Primers used for the PCR reaction on genomic DNA or cDNA. The reactions started at 94°C for 5 min followed by 35 cycles consisting of incubations at 94°C for 30s, 60°C (Tm) for 1 min and 72°C for 1 min. At the end of the cycles phase, the PCR mixtures were incubated at 72°C for 10 min. For each PCR assay, a negative control (amplification mix without DNA) was used to detect possible contamination.
