# Supplementary material for: New global analysis of the microRNA transcriptome of primary tumors and lymph node metastases of papillary thyroid cancer
Source: BMC Genomics. 2015 Oct 21;16:828. doi: 10.1186/s12864-015-2082-3 (PMC4618137; doi:10.1186/s12864-015-2082-3)
Supplement: Additional file 2: — Supplemental materials and methods. (PDF 652 kb) [file 12864_2015_2082_MOESM2_ESM.pdf]

Supplemental materials and methods

- **Genomic DNA extraction**

For each sample, the remaining 1 ml mix of phenol-chloroform was mixed with 0.3 ml of 100% ethanol. After an incubation of 2 minutes at room temperature, samples were centrifuged at 2000g and 4°C during 5 minutes. The pellet was washed 2 times during 30 minutes at room temperature and with periodical agitation. The washed solution consisted of a mix of ethanol 30% with 0.1 M of sodium citrate. After each wash, samples were centrifuged at 2000g and 4°C during 5 minutes. The pellet was suspended in a solution of 2 ml of 75% ethanol during 15 minutes at room temperature with a periodical agitation, then samples were centrifuged again as previously described. After 5 minutes at room temperature, the pellet was suspended in 100 µl of $T_{10}E_1$. Finally, samples were stored at 4°C. Both RNA and DNA concentrations were spectrophotometrically quantified and RNA integrity was verified using an automated gel electrophoresis system (Experion, Biorad).

- **Laser Capture Microdissection**

Frozen sections (4-6µm each) from samples were placed on MembraneSlides 1.0 PEN (ZEISS). Slides were conserved at -20°C in an RNase Free environment during a maximum of 24 hours. Before LCM, they were stained with Cresyl Violet Acetate (ALDRICH), according to ZEISS recommendations. The LCM was performed with a 337 nm PALM MicroLaser System (ZEISS) according to the manufacturer's recommendations. For each sample, an area of about 100 000 $µm^2$ with the highest possible fraction of thyrocytes was dissected in a maximum time of 15 minutes. The dissected samples were collected in the cap of collection Microtubes 500 (415101-4400-200, ZEISS) and immediately mixed with 40 µl of Qiazol (Qiagen). They were stored at -20°C during maximum 24 hours. Total RNA was extracted from dissected tissues with miRNeasy Micro kit (Qiagen) according to the manufacturer's recommendations.

- **qRT-PCR validation of microRNA quantification**

Expression profile experiments: results obtained from bioinformatics analyses of differential expression profiles were validated on both deep-sequenced samples and an independent set of 14 matched normal, tumors and LNM samples. The Universal cDNA Synthesis Kit II (Exiqon) was used to obtain cDNA from total RNA of each sample according to the manufacturer's instructions. A dilution curve was realized in duplicate with ranging dilution points from 20 ng to 200 pg of total RNA from the same sample. Non-template controls (NTC) were also performed. A Pick-&-Mix custom panel (96 wells) with primers for 20 microRNA assays, U6 snRNA assay, and 2 controls from Exiqon (UniSp6 CP and UniSp3 IPC) was designed for the q-PCR experiment. The matched samples from the same patient were analyzed on the same plate. The q-PCR reaction was performed in an AB 7500 HT (Applied Biosystems) with the ExiLENT SYBR Green Master Mix Kit (Exiqon) according to the manufacturer's instructions. A melting curve was performed for each reaction.

Analysis method: the data were first analyzed with the 7500 software and the Ct values were obtained by using automated baseline and threshold settings. All NTC values were undetected. The UniSp3 value of each sample was in the same range. Ct values obtained for the dilution curve were plotted against the log10 of the dilution for each microRNA assay. Curves were obtained using the least squares analysis. The linearity of each assay was identified by visual inspection of the curve and the Ct linear range corresponding to the assay-specific limit of detection was established. Ct outside these ranges were removed. PCR efficiencies were calculated as $10^{(-1/slope)}-1$ for each assay linear range. Relative quantifications (RQ) were calculated based on these efficiencies with the Ct values and the Pfaffl method [67]. Values were normalized between samples based on U6 SnRNA expression. Paired t-test was used to compare the expression values between tissue types.

5p to 3p arm modulation experiments: results from the bioinformatics analyses of 5p-to-3p arm modulations were validated on both samples used for small RNA deep-sequencing and an independent set of 14 matched normal, tumors and LNM samples. Universal cDNA Synthesis Kit

II (Exiqon) or TaqMan MicroRNA Reverse Transcription kit (Applied biosystems) was used to obtain cDNA from total RNA for each sample according to the manufacturer's instructions. For each assay, a dilution curve was performed in duplicate with 10 ranging dilution points from 2000 ng to 8 pg of synthetic microRNA (Eurogentec) designed according to the canonical form given in miRBase v19. A non-template control (NTC) was also performed. Dilution curves and matched samples were analyzed in the same plate for each q-PCR assay. Q-PCR reactions were performed in triplicate in an AB 7500 HT (Applied Biosystems) with the ExiLENT SYBR Green Master Mix Kit and microRNA LNA PCR primers (Exiqon) or with the TaqMan Universal PCR Master Mix and TaqMan MicroRNA Assays probes (Applied biosystems) according to the manufacturer's instructions. A melting curve was performed for each reaction.

Analysis method: the data were first analyzed with the 7500 software and Ct values were obtained using automated baseline and threshold settings. All NTC values were undetected. Ct values obtained for the dilution (standard) curve were plotted against the log10 of the dilution for each microRNA assay. The standard curve was obtained using the least squares analysis. The linearity of each assay was identified by visual inspection of the curve and the Ct linear range corresponding to the assay-specific limit of detection was established. Ct values outside theses ranges were removed. Absolute quantifications (AQ) were calculated based on these curves. For each sample, values obtained corresponding to each mature microRNAs arm were converted to 5p/3p ratios. Statistical significance of ratio modulations was assessed using a paired t-test.

- **MicroRNA A-to-I editing validation and BRAF V600E status**

We screened microRNA editions in genomic DNA and precursor form of microRNA in samples used for deep-sequencing. For the precursor form, we started with total RNA and after a DNase treatment with DNase I (Invitrogen), 1 µg of total RNA of each sample was used to obtain cDNA using hexamers (3.6 µg/µl) (Roche) and Superscript II RNase H Reverse Transcriptase (Invitrogen) according to the manufacturer's instructions.

PCR reactions, for both genomic DNA or cDNA, were performed with the recombining Taq DNA polymerase kit (Invitrogen). Each PCR reaction was performed in the presence of 5 µl 10X PCR

buffer, 1.5 µl $MgCl_2$ (50 mM), 1 µl dNTP mix (10 mM, Invitrogen), 1 µl of the forward and the reverse primer (10 µM each), 0.4 µl Taq DNA polymerase (5U/µl), 2 µl of DNA from the RT reaction and 38.1 µl of distillate water. Primers and PCR conditions are detailed in additional file 2: Table S4. Primers were designed with Primer 3 software (http://bioinfo.ut.ee/primer3/) and their specificity was verified using BLAST alignment (NCBI) on the whole human genome.

PCR products were purified with the ExoSAP-IT (Affymetrix, USB) according to the manufacturer's instructions. Sequencing was performed with the BigDye Terminator V3.1 Cycle Sequencing Kit (Applied Biosystems) with the sequencer ABI PRISM 3130 (Applied Biosystems) and the genetic analysis program 3130-XI. Sequences were analyzed with NCBI BLAST (http://blast.ncbi.nlm.nih.gov/Blast.cgi) and a visual inspection of the electropherograms. In previous studies, this method showed equivalent results compared to TOPO cloning to screen editing in pri-microRNA [68].

- **Human small RNA deep-sequencing: downstream analyses**

Identification of novel microRNA: we added a specific procedure to identify novel -5p or -3p microRNAs (Additional file 2: Figure S8). For each human precursor encoding a single mature microRNA according to miRBase v19, tags from all nine samples mapped to this precursor were pooled together. We further extracted all tags that did not overlap the canonical mature microRNA annotated for this precursor. To determine whether this subset of tags originate from random noise or exhibit a pattern of novel 5p or 3p arm, we computed, for each tag, the number of individual reads that would cluster with it considering the 5 bp windows defined around its mapping coordinates. The tag associated to the highest read count was considered as a novel canonical microRNA if it satisfied the following: (1) a minimum of 2 tags are found across all nine libraries; (2) a minimum of 50 individual reads across all libraries overlap this novel microRNA within the specified windows; (3) at least 60% of mapped individual reads have a length superior or equal to 20 nucleotides; (4) at least 50 % of mapped individual reads are perfect matches. Note that this would imply that the canonical microRNA is always the most represented in terms of individual read count, which is not systematic in practice. Nonetheless,

this approach still offers a good approximation to identify coordinates of unannotated microRNAs and estimate their relative expression.

Characterization of isomiR distribution: in this manuscript, the set of isomiRs for a given mature microRNA includes only variants with shifted coordinates, regardless of nucleotides that were trimmed prior alignment and without considering mismatches in the aligned sequences. Substitutions caused by genomic mutations or RNA-editing and non-templated additions are analyzed separately further in this manuscript.

We characterized isomiR distributions between tissue types. We excluded all mature microRNAs that did not have a minimum TMR (Total Mapped Read) of 100 in all nine samples. For each of the remaining microRNA, we determined the relative contribution of each isomiR i to total expression by: contribution (i, miR) = 100 * read_count(i, miR) / TMR(miR). We considered as "true" isomiR (chr:start-end:strand) elements with a minimum relative contribution of 1 %. Other were considered random noise and discarded prior any other computation. We then calculated the number of distinct isomiRs for each mature microRNA independently for every sample, and averaged the distribution of isomiR numbers per microRNA for each tissue type.

We further characterized isomiR variations. We assigned each isomiR to one of the following classes: canonical (strictly matches miRBase reference microRNA), start-site (5' coordinate is modified), end-site (3' coordinate is modified), or both-sites (both 5' and 3' coordinates are modified) and the relative contributions of each class to total expression were computed independently for each mature microRNA in every sample.

We further tracked the variation of each isomiR across disease progression for every mature microRNA to identify specific isomiR with different contribution between tissue types. We averaged individual isomiR contribution over tissue types (namely normal, tumor and LNM) and screened all mature microRNAs to detect specific isomiRs showing a mean variation of 20% or more of the total mapped reads when comparing contribution in normal tissues to primary tumors or metastases.

Characterization of the 3' non-templated additions (NTA) distribution: we analyze additions occurring on both canonical microRNAs and their relative isomiRs while limiting cross-mapping effects. We collected for each sample the set of tags mapped only during the first alignment step (perfect matches, no mismatch allowed) and overlapping canonical microRNA within a [±5bp, ±5bp] window. We then counted, for each individual microRNA, the number of reads harboring 3' NTA and tabulated read counts per type of addition. We further excluded all microRNAs for which the number of mapped reads was below 100, leading to a subset of 189 microRNAs.

We first attempted to distinguish true non-ambiguous additions from background errors due to incorrect adaptor trimming or sequencing errors. We evaluated the individual contribution of each 3'addition for every selected microRNA. The mean contribution was further obtained by averaging across all microRNAs and samples. Only nucleotides accounting for 1% or more of all additions were considered significant.

We performed this calculation after mapping reads to both human precursors and the complete reference genome. For precursors alignment, we identified a total of 3046 types of additions accounting for less than 1% of all additions across all samples. For whole genome alignment, for which a maximum of 2 nucleotides were trimmed, only 12 types of additions with a contribution below 1% were identified. Nonetheless, 8 types of additions above 1% contribution were the same for both approaches and their ranking was not modified. Our current hypothesis is that all these artefactual additions result from random sequencing errors, inaccurate adaptor trimming or incorporation of incorrect bases during adapter ligation. We further computed the contribution of each type of addition for every sample independently by averaging contributions across all microRNAs.

Since prevalent additions consisted of A and U additions (A, AA, U, UU), we then established amounts of adenylation and uridylation for each microRNA. Fraction of adenylated and uridylated reads was obtained by summing all reads harboring one or several A or U additions divided by the total number of mapped reads for each microRNA.

Finally, we screened every microRNA for variations of adenylation or uridylation across the course of the disease. We required a variation of at least 20% of the total mapped reads, consistent for each of the three samples, when comparing normal tissues to primary tumors or metastases.

Identification of single nucleotide substitutions and microRNA editing: single nucleotides were identified using a custom approach based on pileup alignment. Since read depth could reach more than thousands of reads for certain microRNAs, we first screened all covered genomic positions using alignments collapsed by read sequence to reduce computational efforts. Only positions overlapping microRNAs precursors were considered. If at least one base different from the reference was found at a given position, tags were uncollapsed to extract individual read count information and compute the true allelic fraction of the alternate base at this position. We further required that a variant: (1) had a minimum read depth of 20, (2) had a minimum allelic fraction of 15 %, (3) had less than 75 % of its supporting alternate bases located in the first or two last positions of the read. After running this variant calling pipeline on every sample and removing known SNPs and putative false positives, we obtained a list of edited microRNAs.

- **Confirmation with TCGA public microRNA deep-sequencing thyroid cancer data**

We validated our small RNA deep-sequencing expression profiles on additional human thyroid samples. We collected public data from The Cancer Genome Atlas (TCGA) (https://tcga-data.nci.nih.gov/tcga/). We used the firehose_get utility version 0.4.3 (https://confluence.broadinstitute.org/display/GDAC/Download) to download public data from the Broad Institute's Genome Data Analysis Center (GDAC). Processed analyses available from the Broad GDAC only include primary tumors. We downloaded directly preformatted data tables invoking firehose_get with the following command line: firehose_get –t clinical miR stddata latest THCA

With the above command line, we retrieved the latest data tables for both clinical and microRNA expression profiles for all thyroid samples available at the TCGA. Note that these data tables included information for every thyroid sample type: primary solid tumor (TP, code 01), metastatic (TM, code 06) and solid normal tissue (NT, code 11). Identification of sample type and pairing between matched normal-tumor and matched tumor-metastasis was obtained from the TCGA barcode associated to each sample.

We also retrieved information about percent tumor nuclei and percent tumor necrosis. Since this information is only available in the *biospecimen* data files, which are not included in the Broad GDAC preformatted data tables, we downloaded them directly from the TCGA Data Portal (https://tcga-data.nci.nih.gov/tcga/tcgaDownload.jsp) using the Data Matrix web utility. The association between sample, percent tumor nuclei and percent tumor necrosis was also obtained from TCGA barcodes. We obtained clinical information and BRAF V600E mutational status from the supplementary information of the TCGA article: Table S2 [30].

microRNA expression tables obtained from the Broad GDAC were available in two distinct forms: a first table containing precursor expression and a second table listing the expression of each individual mature isoform (based on hg19 coordinates). Additionally, annotation of mature isoforms in TCGA tables was still based on the *miR/miR** nomenclature, while our bioinformatics pipeline was based on miRBase v19 in which mature microRNA had the *-5p/-3p* suffixes. To obtain mature microRNA expression from TCGA data with the updated -*5p/-3p* annotation, we designed an in-house R script to reshape TCGA data tables and obtain expression values for each mature microRNA using the same miRBase reference annotation as we used for our own microRNA sequencing data (Additional file 2:Figure S9). Briefly, each mature microRNA from miRBase v19 was converted to GenomicRanges data structures based on chromosome, strand, start and end coordinates information. The name of the mature microRNA (e.g. hsa-miR-146b-5p) was also used as additional metadata. Similarly, all reported isomiR variants in downloaded TCGA table were converted to GenomicRanges data structures. We then used the findOverlaps function to associate to each TCGA isoform the corresponding mature

microRNA reported in miRBase. To closely mirror our definition of isomiRs, which must be located within a window of ±5bp around their corresponding canonical form, we enlarged the GenomicRanges corresponding to mature microRNAs from miRBase by respectively subtracting and adding 5 nucleotides to their start and end coordinates. The findOverlaps function was then called with the additional argument type='within' to remove TCGA isoforms extending outside this specified window. Expression profile matrices were further obtained by summing expression values (in Count Per Million) of every isomiR variant for a given (TCGA barcode, mature microRNAs) pair. This ensured that expression values for a given mature microRNA in TCGA samples were obtained using all overlapping mapped reads, similar to the strategy employed in our own bioinformatics analysis. If a given (TCGA barcode, mature microRNAs) pair was not found in TCGA table, we assumed that the corresponding CPM was 0.

The expression matrix (562 samples including 59 normal thyroid tissues, 495 primary tumors and 8 lymph node metastases) was further filtered to include all normal thyroid tissues and primary tumors using the following:

- only classical papillary thyroid cancers were used (*patient.histologicaltype = thyroid papillary carcinoma - classical/usual* in clinical data)
- only tumors classified as N1 based on TNM cancer staging were kept (*patient.stageevent.tnmcategories.pathologiccategories.pathologicn = n1, n1a, n1b or n1x* in clinical data)
- a minimum of 70 % of tumor nuclei was required (*tumor_nuclei_percent ≥ 70* in biospecimen data)
- a maximum of 20 % of necrosis was allowed (*tumor_necrosis_percent ≤ 20* in biospecimen data)

leading to a total of 120 primary tumors. We then evaluated microRNA expression levels for each tissue type using filtered and unfiltered TCGA thyroid samples. Significance was assessed using unpaired t-test on CPM values when comparing two tissue types. In addition, we analyzed the differences in the microRNA expression levels between clinical sub-populations of the tumor

samples determined with the supplementary information of the TCGA article: Table S2 [30]. Principal components analyses were performed with "PRcomp" in R.

Reformatted TCGA data were also used to evaluate 5p-to-3p expression ratios, and a similar methodology was used to analyze isomiR distributions. This time, each individual TCGA isoform was first attached to its overlapping miRBase mature microRNA, as previously described, but CPM values were not summed by (microRNAs, TCGA barcode) pair. Instead, the contribution of each isomiR was evaluated for every (microRNAs, TCGA barcode) pair, and isoforms with a relative contribution below 1 % were removed. The distribution of the number of isoforms per microRNA was obtained by averaging the number of isoforms per microRNA across tissue types. A chi-squared test was used to determine whether this distribution was different when comparing normal tissues to primary tumors and metastases.

References

67 Pfaffl MW: **A new mathematical model for relative quantification in real-time RT-PCR.** *Nucleic Acids Res* 2001, **29**:e45.

68 Kawahara Y: **Quantification of adenosine-to-inosine editing of microRNAs using a conventional method.** *Nat Protoc* 2012, **7**:1426–37.
